# Characterization of novel bangle lectin from *Photorhabdus asymbiotica* with dual sugar-binding specificity and its effect on host immunity

**DOI:** 10.1371/journal.ppat.1006564

**Published:** 2017-08-14

**Authors:** Gita Jančaříková, Josef Houser, Pavel Dobeš, Gabriel Demo, Pavel Hyršl, Michaela Wimmerová

**Affiliations:** 1 Central European Institute of Technology (CEITEC), Masaryk University, Brno, Czech Republic; 2 National Centre for Biomolecular Research, Faculty of Science, Masaryk University, Brno, Czech Republic; 3 Department of Animal Physiology and Immunology, Institute of Experimental Biology, Faculty of Science, Masaryk University, Brno, Czech Republic; 4 Department of Biochemistry, Faculty of Science, Masaryk University, Brno, Czech Republic; Stanford University, UNITED STATES

## Abstract

*Photorhabdus asymbiotica* is one of the three recognized species of the *Photorhabdus* genus, which consists of gram-negative bioluminescent bacteria belonging to the family *Morganellaceae*. These bacteria live in a symbiotic relationship with nematodes from the genus *Heterorhabditis*, together forming a complex that is highly pathogenic for insects. Unlike other *Photorhabdus* species, which are strictly entomopathogenic, *P*. *asymbiotica* is unique in its ability to act as an emerging human pathogen. Analysis of the *P*. *asymbiotica* genome identified a novel fucose-binding lectin designated PHL with a strong sequence similarity to the recently described *P*. *luminescens* lectin PLL. Recombinant PHL exhibited high affinity for fucosylated carbohydrates and the unusual disaccharide 3,6-*O*-Me_2_-Glcβ1–4(2,3-O-Me_2_)Rhaα-*O*-(*p*-C_6_H_4_)-OCH_2_CH_2_NH_2_ from *Mycobacterium leprae*. Based on its crystal structure, PHL forms a seven-bladed β-propeller assembling into a homo-dimer with an inter-subunit disulfide bridge. Investigating complexes with different ligands revealed the existence of two sets of binding sites per monomer—the first type prefers l-fucose and its derivatives, whereas the second type can bind d-galactose. Based on the sequence analysis, PHL could contain up to twelve binding sites per monomer. PHL was shown to interact with all types of red blood cells and insect haemocytes. Interestingly, PHL inhibited the production of reactive oxygen species induced by zymosan A in human blood and antimicrobial activity both in human blood, serum and insect haemolymph. Concurrently, PHL increased the constitutive level of oxidants in the blood and induced melanisation in haemolymph. Our results suggest that PHL might play a crucial role in the interaction of *P*. *asymbiotica* with both human and insect hosts.

## Author summary

*Photorhabdus asymbiotica*, originally isolated in 1989 from a patient in the USA, was revealed to be not only effective insect pathogen but also bacteria causing serious and difficultly treatable diseases in humans. The genome of *P*. *asymbiotica* is being intensively studied but little is known about the details of the host-pathogen interaction at the molecular level. In this paper, we focused on the lectin identified in *P*. *asymbiotica* genome that may be crucial for the early stage of infection. We designated this lectin as PHL and prepared it in fully functional recombinant form. We identified its specificity and investigated the structural details of ligand binding. We found the PHL structure unique on the basis of number and arrangement of binding sites present in each monomer. Furthermore, we investigated influence of PHL on immune system of human and insect to propose the biological role for this lectin. The antimicrobial activity as well as phenoloxidase activity and reactive oxygen species production which contribute to antimicrobial effect was modulated after exposition to PHL in both blood and haemolymph supporting the idea that PHL might play important role in the host-pathogen interaction.

## Introduction

*Photorhabdus* is a genus of three species belonging to the gram-negative entomopathogenic bacteria of the family *Morganellaceae*. Unlike the other two species of the genus, *P*. *asymbiotica* is not only an insect pathogen. Using a still poorly understood mechanism, *P*. *asymbiotica* can infect humans and cause both locally invasive soft tissue infection and disseminated bacteraemic disease characterised by multifocal skin and soft tissue abscesses [1–4]. While other members of the genus are not able to replicate and survive above 32–34°C, *P*. *asymbiotica* has the ability to grow at temperatures above 37°C [4–6]. *P*. *asymbiotica* can be further subdivided into two apparent subspecies—American and Australian isolates according to genotypic criteria and the occurrence of human infection. In general, it was found that Australian strains are more virulent than American ones [3,7,8].

The life cycle of *Photorhabdus* as an insect pathogen is well-characterized [2,9]. *Photorhabdus* does not exist in a free-living form in the soil, but engages in a specific mutualistic association with entomopathogenic nematodes (EPN) of the genus *Heterorhabditis*. This nematobacterial complex is highly pathogenic for a broad range of insects. Using EPN as a vector, *Photorhabdus* bacteria cells are delivered into the haemocoel of insect larvae, where they are regurgitated by the nematodes and kill the host within 48 h by a combination of the toxins’ action and septicaemia [3,10]. The cadaver serves as a nutrient source for both the bacterial pathogen and developing nematodes. Subsequently, the bacteria and new infective juvenile nematodes re-associate and search for a new host. In humans, *P*. *asymbiotica* employs a so-called “nutritional virulence” strategy—it aggressively acquires amino acids, peptides and other nutrients from the host [5]. Previous studies revealed that many of the cytotoxins and virulence factors produced by *Photorhabdus* are equally effective against both insect and mammalian immune defence mechanisms [5,11]. The treatment of reported cases has required extensive antibiotic intervention with relapses in many cases [1,4]. It is interesting to note that *P*. *asymbiotica* is not the only bacterial symbiont of nematodes associated with human diseases. *Wolbachia*, an endosymbiont of the nematodes *Onchocera volvulus* and *Brugia malayia* that cause river blindness and lymphatic filariasis, was reported to stimulate human immune system via production of endo-toxins that are released from nematodes upon death or damage [1,12–14]. Compared with *P*. *asymbiotica*, *Wolbachia* is not capable of active reproduction in the human host, which makes *P*. *asymbiotica* a potentially more dangerous pathogen [15]. There is also a close phylogenetic relation to *Yersinia pestis*, a cause of plague, and the parallel between their behaviour is noteworthy [3,5].

Pathogenic bacteria often use lectins, i.e. protein receptors with a high specificity for glycoconjugates, to recognize and adhere to human tissues [16,17]. In general, lectins are ubiquitous carbohydrate-binding proteins, which play a crucial role in many physiological and pathophysiological processes [18]. Different affinity towards various ligands enables lectins to “read” the information stored in carbohydrate molecules, thus making them a powerful tool in cell-cell recognition, immunity, cancer, pathogen adhesion, etc. [19,20]. Recently, a new fucose-binding lectin PLL was identified in *P*. *luninescens* and structurally characterized [21].

This article describes the identification, cloning and production of a novel recombinant l-fucose/d-galactose-binding lectin from *P*. *asymbiotica* (designated PHL). The interaction of PHL with carbohydrate ligands was analysed through biophysical methods, and the structures of PHL and its complexes with saccharides were solved. The ability of PHL to act as a host-cell recognizing agent was investigated through interaction with the haemolymph of *Galleria mellonella* (order Lepidoptera, family Pyralidae) and human blood components.

## Results

### Identification and purification of PHL

**Fig 1:**
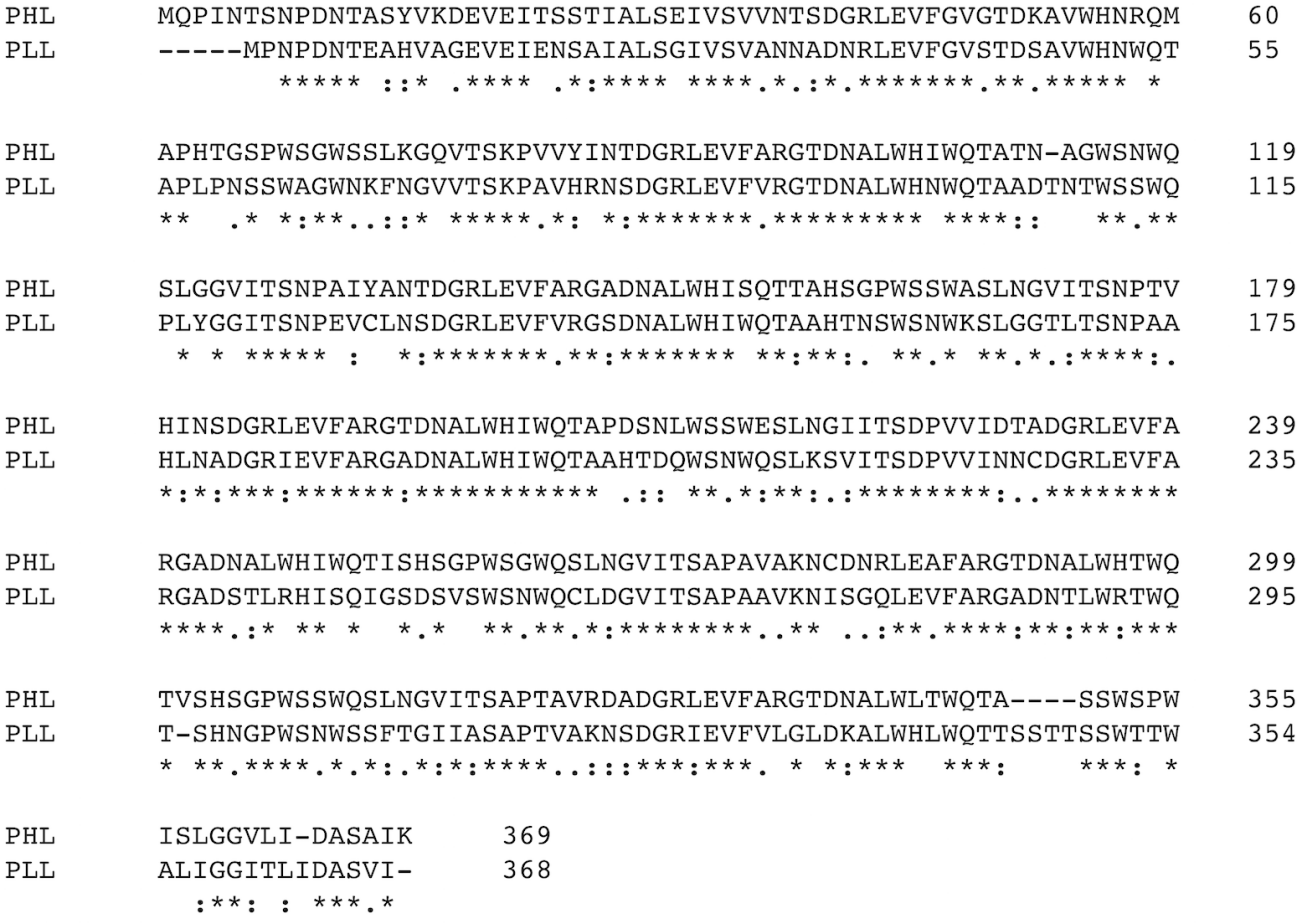
Sequence alignment of PHL (NCBI Reference Sequence: WP_012776886.1) and PLL (Sequence ID: 5C9L_A) proteins. The asterisk indicates fully conserved residues, the colon a strong conservation between groups of amino acids and the dot is for weak similarity.

PHL was identified as a PLL-like protein in the translated genome of *Photorhabdus asymbiotica* (strain ATCC 43949). The protein consists of 369 amino acids with 72% similarity to PLL (63% identity) (Fig 1).

The synthetic *phl* gene was cloned and overexpressed in *E*. *coli*. The recombinant protein was isocratically purified using single-step affinity chromatography on a mannose-agarose column, and its purity was verified by SDS-PAGE. The protein was confirmed to be PHL (40.18 kDa monomer) by MALDI-MS/MS.

### Carbohydrate specificity of PHL

**Fig 2:**
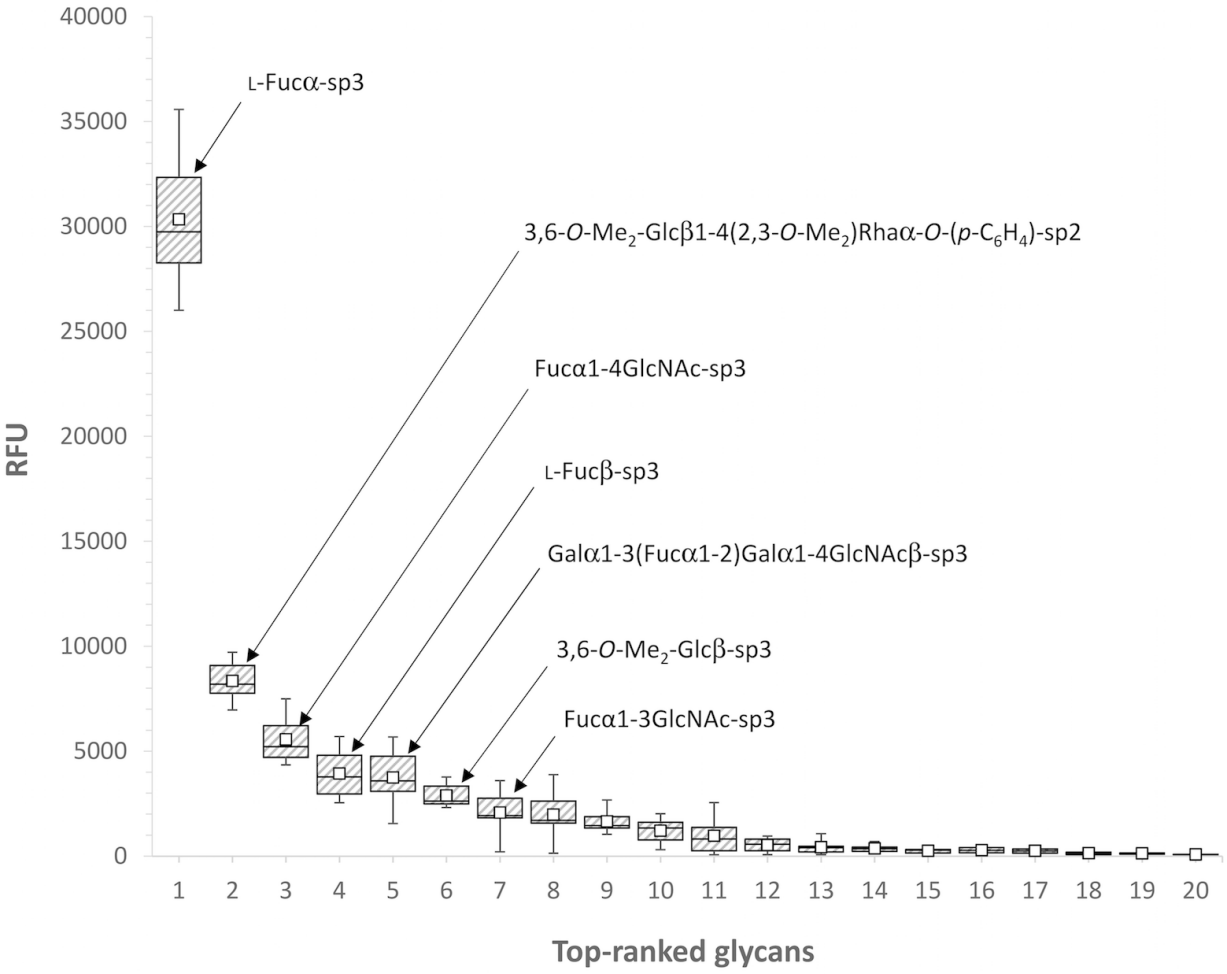
Box and whisker plot for glycan array screening for PHL (200 μg/ml) labeled with DyLight 488 NHS Ester. The top 19 saccharides and control trehalose (sample 20) were selected for display. The 7 saccharides giving the highest average signals are depicted. Linker formula—sp2: -O-CH_2_CH_2_NH_2_; sp3: -O-(CH_2_)_3_NH_2_. The bottom and top of the box are the first and third quartiles; the band inside the box is the second quartile (the median); the ends of the whiskers represent the minimum and maximum values of the data and the small squares inside the boxes represent the mean. RFU, relative fluorescence units. Complete glycan array results and the raw data are given in S1 and S2 Tables, respectively.

A screening of PHL binding to various glycans was performed using glycan array microchips containing over 600 different mammalian glycans, bacterial polysaccharides, glycosylated peptides and proteins (S1 and S2 Tables). PHL recognized 12 saccharides with at least a 5-fold higher response than that with trehalose (standard blank, sugar ID 629). The lectin was found to be specific mainly towards fucose and oligosaccharides containing terminal fucose residues as shown in Fig 2. PHL displays a considerable preference for α-fucoside, but β-fucoside is also strongly recognized. The most preferred complex saccharide was 3,6-*O*-Me_2_-Glcβ1-4(2,3-*O*-Me_2_)Rhaα-*O*-(*p*-C_6_H_4_)-OCH_2_CH_2_NH_2_, an unusual disaccharide present in the *Mycobacterium leprae* glycolipid PGL-I [22,23]. PHL displayed a preference for short fucosylated glycans, as is demonstrated by its preference towards Fucα1-4GlcNAc and Fucα1-3GlcNAc disaccharides over the complex saccharides, e.g. whole Lewis antigens. The strongest binding to human-related oligosaccharides is observed towards blood group B trisaccharide.

**Fig 3:**
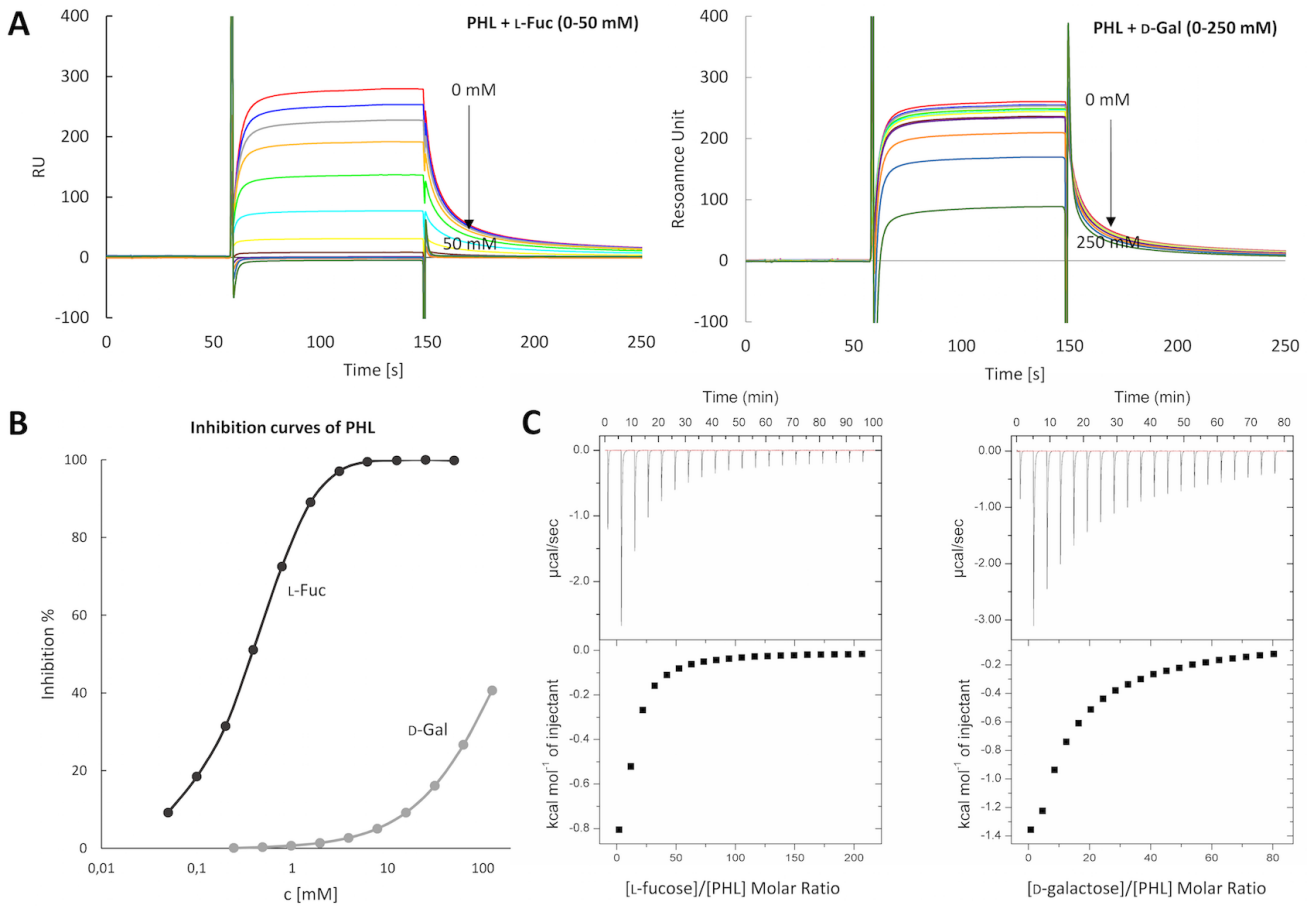
Determination of carbohydrate specificity/affinity of PHL towards l-fucose and d-galactose. **(A)** SPR sensorgrams (differential curves) displaying PHL binding (0.25 μM) to CM5 sensor chip with immobilized l-fucoside in the presence of competing saccharides. Response decreases with increasing concentration of the inhibitor–l-fucose (0–50 mM), d-galactose (0–250 mM). **(B)** A logarithmic plot of inhibition curves calculated from SPR measurements **(C)** ITC curves of PHL (50 μM) titration by l-fucose (20 mM) and d-galactose (50 mM). 20 injections of 2.0 μl of sugars were added every 240 s to a PHL-containing cell. Lower plots show the total heat released as a function of total ligand concentration for the titration shown in the upper panels.

The Surface Plasmon Resonance (SPR) technique was employed to further analyze the specificity/affinity of PHL towards various sugars. It was revealed that PHL interacts with the immobilized α-l-fucoside, giving an apparent K_D_ of 1.4±0.21 μM, whereas no visible binding to d-mannoside and d-galactoside was observed. Its competitive inhibition with seven monosaccharides (l-Fuc, Me-α-l-Fuc, Me-β-l-Fuc, d-Man, d-Gal, d-Glc, d-GlcNAc) and five oligosaccharides (Fucα1-3GlcNAc, Fucα1-4GlcNAc, blood group H, A and B trisaccharides) were also tested (Table 1, Fig 3A and 3B). The lowest IC_50_ was determined for Me-α-l-Fuc and blood group B trisaccharide, being about 10 and 5 times stronger inhibitors than free l-fucose, respectively.

**Table 1:** Inhibitory effect of representative carbohydrates on PHL binding to immobilized l-fucoside. IC_50_ was determined from a plot of serial dilutions vs % inhibition, and potency was relative to l-Fuc.

Ligand	IC_50_ [mM]	Potency
Me-α-l-Fuc	0.04	9.39
BGB trisaccharide	0.07	5.15
BGA trisaccharide	0.30	1.21
**l****-Fuc**	**0.36**	**1.00**
Fucα1-3GlcNAc	0.69	0.53
Fucα1-4GlcNAc	0.73	0.49
Me-β-l-Fuc	0.77	0.47
BGH trisaccharide	1.09	0.33
d-GlcNAc	70.50	0.01
d-Man	249.53	< 0.01
d-Glc	342.45	< 0.01
d-Gal	377.86	< 0.01

Its binding to a range of saccharides was further characterized by isothermal titration calorimetry (ITC), enabling determination of the complete thermodynamic profile of the molecular interaction (Fig 3C). The calculated dissociation constants (Table 2) are in the millimolar range, indicating a low affinity to the chosen carbohydrate ligands, as is usually observed for lectin/saccharide interactions. Me-α-l-Fuc, blood group B trisaccharide and d-Gal were revealed to be stronger binders than l-Fuc (K_D_ is in submillimolar values). The highest affinity was determined towards Me-α-l-Fuc, with a K_D_ 5 times lower than l-Fuc. With Me-α-l-Fuc and the blood group B trisaccharide, the equilibrium dissociation constants are 0.27 mM and 0.49 mM, with binding stoichiometry of approx. 2.9 and 4.3, respectively. However, the stoichiometry value *n* cannot be properly calculated, and especially for other ligands, due to the low affinity. Therefore, the stoichiometry was fixed during the fitting procedure to 3 and 4, respectively, which enabled a comparison of individual sugars (Table 2).

**Table 2:** Equilibrium dissociation binding constants for interaction between PHL and carbohydrate ligands determined by isothermal titration calorimetry at 25°C (standard deviations were calculated from three independent measurements).

Ligand	K_D_ [mM] (n = 3)	K_D_ [mM] (n = 4)
Me-α-l-Fuc	0.26±0.04	0.21±0.00
BGB trisaccharide	0.57±0.01	0.51±0.01
d-Gal	0.89±0.02	0.83±0.02
l-Fuc	1.40±0.03	1.33±0.04
BGA trisaccharide	1.45±0.03	1.38±0.04
Fucα1-3GlcNAc	3.52±0.21	3.43±0.21
Me-β-l-Fuc	3.55±0.09	3.48±0.09
d-Glc	3.55±0.15	3.48±0.15
BGH trisaccharide	4.44±0.19	4.35±0.19
Fucα1-4GlcNAc	4.46±0.31	4.37±0.31
d-Man	44.6±2.0	44.4±2.0
d-GlcNAc	ND*	ND*

*ND—not determined

### Oligomeric state of PHL in solution

**Fig 4:**
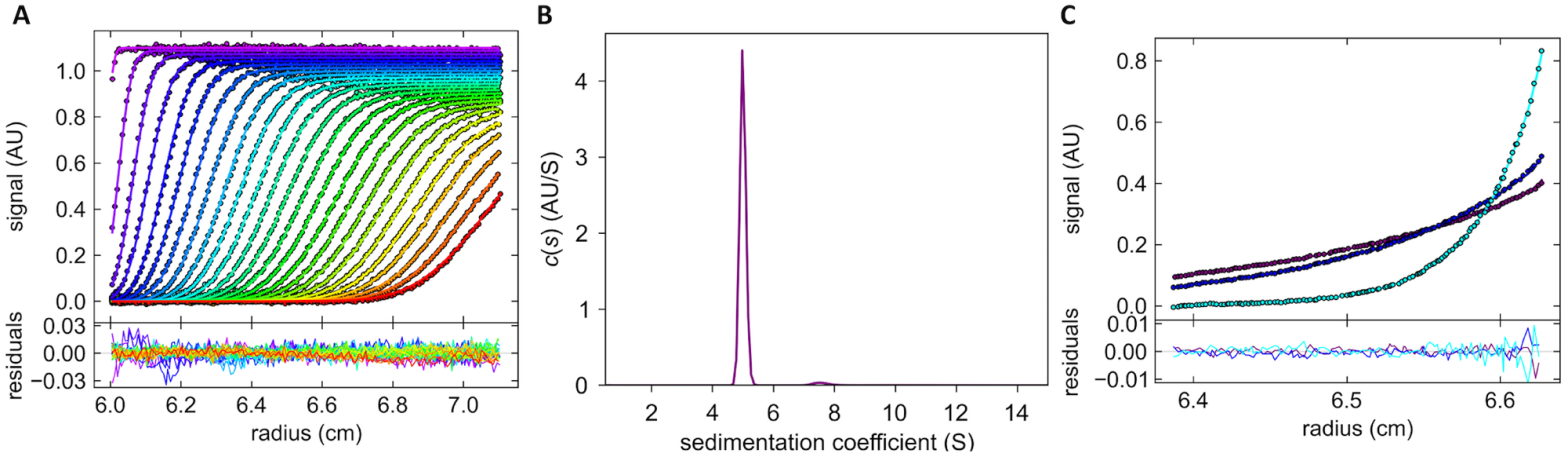
**(A, B). Sedimentation velocity experiment.** Sedimentation profiles and fitted curves of PHL (0.12 mg/ml) in 100 mM NaCl, 20 mM Tris/HCl, pH 7.5 obtained from continuous c(s) analysis using Sedfit (A, upper panel). The experiment was carried out at 42,000 rpm at 20°C, and the scans were recorded every 6 minutes. For simplicity, every third scan is shown. Residual plot (A, lower panel) shows the differences between experimental data and fitted curves, thus reflecting the quality of the fit. The continuous size distribution of sedimenting species (B) for PHL resulted in a peak with a sedimentation coefficient of 4.98 S (s20, w = 5.12 after extrapolation to standard conditions in Sednterp). The value is clearly much higher than the predicted maximum value for a spherical monomer (4.33 S as calculated in Sednterp), suggesting that a dimer with a moderately elongated shape (frictional ratio of 1.34) is formed. The figures were created in the program GUSSI 1.0.8 [24]. **(C) Sedimentation equilibrium experiment.** Equilibrium distributions of PHL (0.05 mg/ml) were obtained at 20°C at rotor speeds of 9,500 (purple curve), 11,400 (dark blue) and 20,000 rpm (light blue). The residual plot (lower panel) shows the goodness of the fit. A global analysis of multi-speed experiments (for all loading concentrations of PHL) using SEDPHAT gave a molecular weight of 78.6 kDa. This value corresponds well with the theoretical molecular weight of the dimer (80.1 kDa). The figure was created in GUSSI 1.0.8.

The determination of the oligomeric state of the PHL lectin was carried out via both techniques of analytical ultracentrifugation (AUC)–sedimentation velocity and sedimentation equilibrium. The continuous size distribution of sedimentation profiles for PHL resulted in a peak with a sedimentation coefficient of 4.98 S suggesting that a PHL exists as a dimer (Fig 4A and 4B). This result was also supported by sedimentation equilibrium experiment (Fig 4C). A global analysis of multi-speed experiments gave a molecular weight of 78.6 kDa what corresponds well with the theoretical molecular weight of the dimer (80.1 kDa). In solution, PHL forms a homo-dimer in contrast to the homologous PLL from *P*. *luminescens*, which exists as a tetramer [21].

### Structure determination

**Fig 5:**
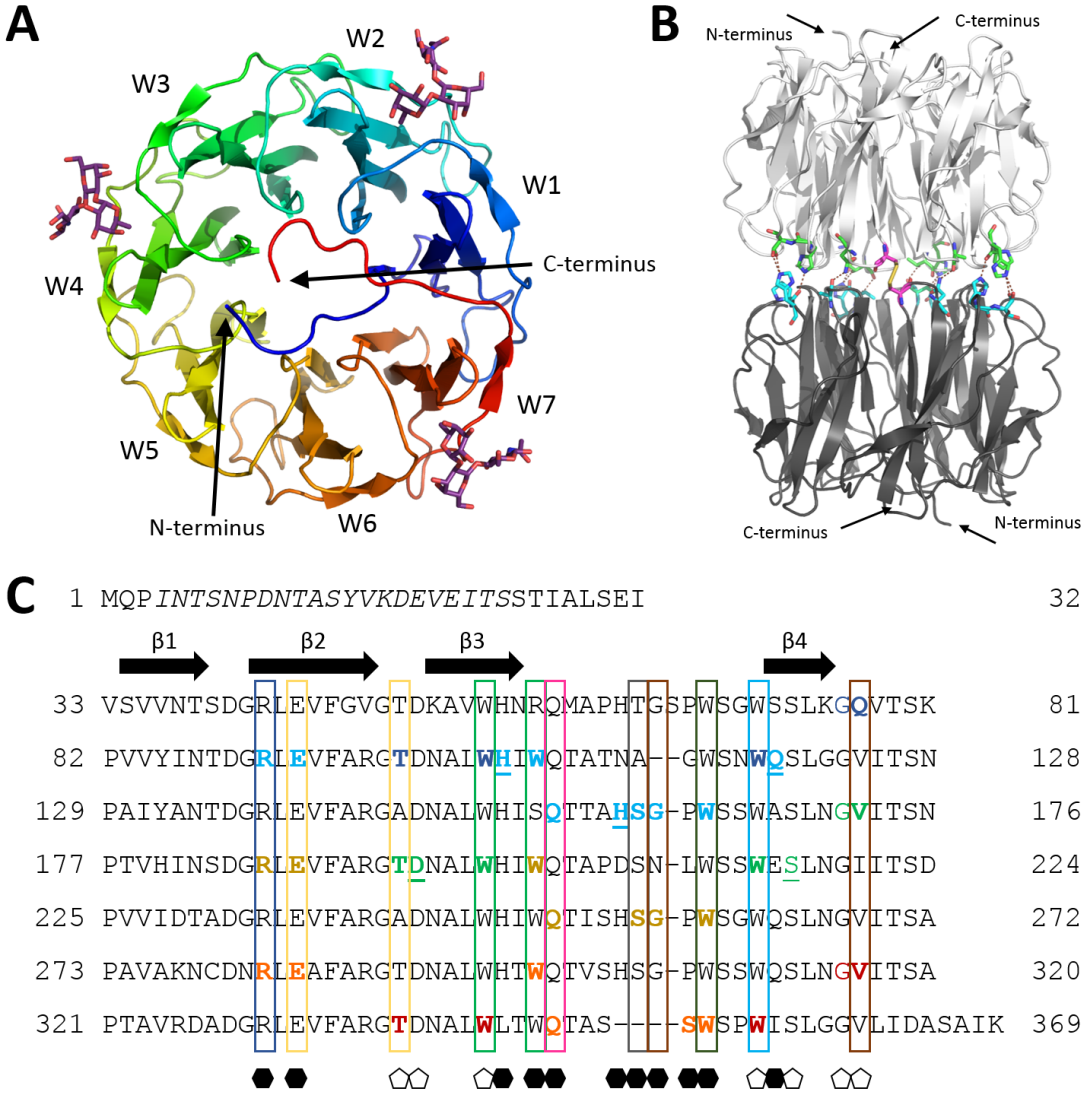
**(A) PHL monomer (chain A) overall architecture with individual blades labelled.** BGH trisaccharide molecules in sites 1F (between blades W1 and W2), 3F (between W3 and W4) and 6F (between W6 and W7) are shown as sticks. **(B) Side view of PHL dimer with intermonomer contacts shown**. Participating residues coloured in green for one monomer and cyan for the second monomer. Disulfide bridge formed by Cys279 is highlighted in magenta. **(C) Alignment of PHL sequence repeats.** Full arrows indicate individual β-sheets. Boxes highlight the conserved residues participating in ligand binding, the colours of the boxes correspond to the residue colouring in Fig 6A. Residues stabilizing Fuc ligands are indicated with an empty pentagon under the column, residues binding Gal ligands are indicated with a full hexagon under the column. Coloured residues in bold bind the corresponding monosaccharide, additional interactions with the oligosaccharide are shown in coloured regular font. The underlined residues only participate in binding through the water bridge. The colour of the residue stands for individual binding sites: site 1F (blue), site 2G (cyan), site 3F (green), site 4G (dark yellow), site 6G (orange), site 6F (red). Residues in italics were not resolved in any structure.

PHL forms a single domain structure, which exhibits a seven-bladed β-propeller fold organized around a seven-fold pseudoaxis of symmetry (Fig 5A). Each repeat of the PHL lectin (W-motif) consists of a four-stranded antiparallel β-sheet connected by relatively long loops. Superposition of the seven β-blades for the PHL lectin gives an overall RMSD value no larger than 0.63 Å. The shape of the PHL lectin monomer is a short torus, with a diameter of 45 Å and a height of 30 Å. The tunnel in the center broadens from 13 Å at the C- and N- termini side to a diameter of 18 Å on the opposite side. The data-collection and refinement statistics are given in Table 3. The overall structure of the PHL monomer is very similar to the recently determined structure of a homologous PLL lectin, with a backbone RMSD of 0.65 Å [21].

**Table 3:** Data collection and refinement statistics. Values in parentheses correspond to highest resolution shell.

	PHL	PHL + Me-α-l-Fuc	PHL + d-Gal	PHL + BGH
PDB ID	5MXE	5MXF	5MXH	5MXG
Beamline	-	BESSY 14.1	BESSY 14.3	BESSY 14.3
Wavelength (Å)	1.541	0.918	0.895	0.895
Space group	P3_1_2(1)	P3_1_2(1)	P3_1_2(1)	P3_1_2(1)
Unit-cell parameters				
a and b (Å)	81.24	81.25	81.32	80.31
c (Å)	113.44	113.23	114.18	112.97
Resolution range (Å)	44.16–1.90 (2.00–1.90)	44.11–1.90 (2.00–1.90)	44.35–1.95 (2.06–1.95)	43.85–2.20 (2.32–2.20)
Reflections measured	124240 (11678)	372940 (54844)	360110 (52064)	271521 (39398)
Unique reflections	34331 (4583)	34705 (4975)	32451 (4636)	21984 (3157)
Completeness (%)	98.8 (92.1)	100.0 (100.0)	100.00 (100.00)	100.0 (98.0)
CC1/2	99.7 (93.4)	99.9 (94.5)	99.9 (87.7)	99.9 (88.5)
R_merge_^†^	0.071 (0.237)	0.076 (0.457)	0.099 (0.790)	0.085 (0.742)
Multiplicity	3.6 (2.5)	10.7 (11.0)	11.1 (11.2)	12.4 (12.5)
<I/σ (I) >	14.7 (4.3)	22.4 (5.3)	18.8 (3.2)	23.8 (3.8)
Reflections used	32591	32917	30740	20916
Reflections used for Rfree	1702	1746	1655	1035
R factor^‡^ (%)	16.64	16.71	17.88	18.53
R_free_^‡^ (%)	19.11	19.58	21.72	22.84
R.m.s.d. bond lengths (Å)	0.009	0.009	0.013	0.012
R.m.s.d. bond angles (°)	1.31	1.30	1.49	1.51
No. of water molecules	363	303	271	103
No. of non-H atoms (total)	3048	3025	3016	2881
Ramachandran plot				
Residues in most favorable regions (%)	97.4	96.8	95.9	93.9
Residues in allowed regions (%)	2.6	3.2	4.1	6.1

^†^ R_merge_ = ∑I_i_—<I>│/∑I_i_, where I_i_ is the intensity of observation and <I> is the mean value for that reflection.

^‡^ R factor = ∑ ││F_o_(h)│—│F_c_(h)││/ ∑_h_ F_o_(h), where F_o_ and F_c_ are the observed and calculated structure-factor amplitudes, respectively. Ser(351) for native and M-Fuc outlier ramachandran.

### Oligomeric state of PHL in crystal

The dimeric state of PHL shown by AUC was also seen in the crystal (Fig 5B). A pseudo-2-fold axis of symmetry generates the dimer associated in a “tunnel to tunnel” manner, so that the C- and N-termini are exposed to the solvent. The main dimer-stabilizing element is a disulfide bridge formed between the Cys279 residues of both monomers in the loop interconnecting β-strands in repeat W6 (Fig 5B). Additional hydrogen bonds are formed by residues in the loops of all repeats except W5.

### Two types of PHL binding sites

The analysis of PHL/Me-α-l-Fuc and PHL/BGH complexes revealed fucose-binding sites located between individual blades of the β-propeller in the upper half of the monomer, where the N- and C- termini are located (Fig 5A). Based on the sequence alignment of individual repetitions, there may be up to seven potential fucose-binding sites per PHL monomer forming a circle (Fig 5). They are referred to as site 1F (between blades W1 and W2), site 2F (between W2 and W3) and so on. In the crystal structure of PHL/Me-α-l-Fuc, which was solved after soaking ligand-free PHL crystals with Me-α-l-Fuc, only sites 3F and 6F were occupied by the ligand.

**Fig 6:**
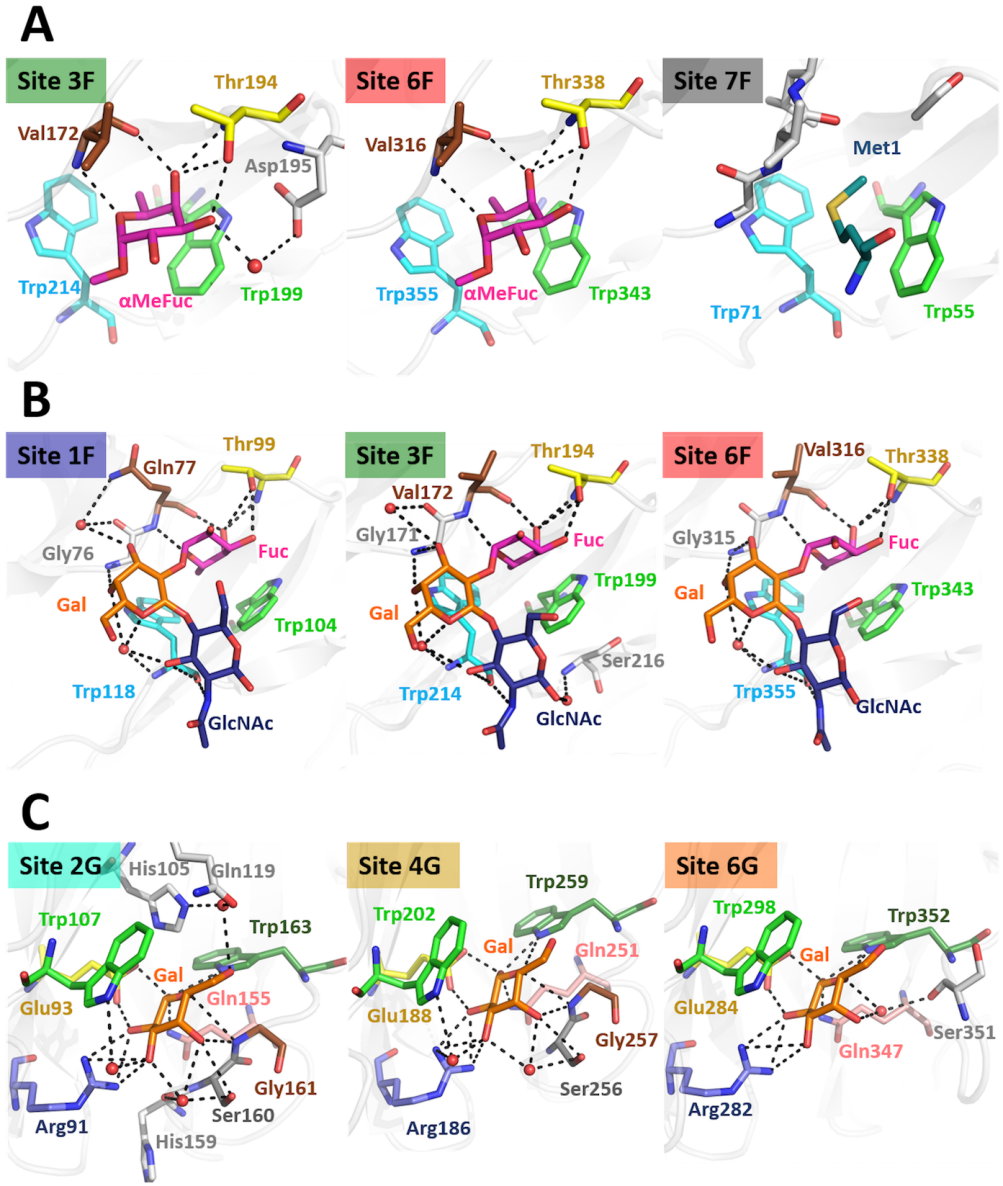
PHL binding sites of PHL/Me-α-l-Fuc complex (A), PHL/BGH complex (B) and PHL/d-Gal complex (C) with the individual ligands. Only sites discussed in the text are shown. Site name colour corresponds to residue colouring in Fig 5C. Residues participating in ligand binding are highlighted with a colour code corresponding to the colour of the boxes in Fig 5C. Saccharide residues are coloured accordingly: Fuc—magenta, Gal—orange, GlcNAc—dark blue. Met coordinated in site 7F shown in teal. Water molecules involved in sugar binding shown as red spheres, hydrogen bonds formed as black dashed lines.

At site 3F (Fig 6A) Fuc-O3, Fuc-O4 and Fuc-O5 atoms are coordinated by the Thr194 side chain and backbone atoms of Thr194 and Val172. The side chains of Trp199 and Trp214 form a hydrophobic pocket which adopts the C6 methyl group of Fuc. Trp199 also forms CH-π interactions with the hydrophobic surface of Fuc C3, C4 and C5. Analogously, hydrogen bonds are formed by Val316 and Thr338, while Trp343 and Trp355 stabilize the hydrophobic part of the saccharide in site 6F (Fig 6A).

Soaking with blood group H trisaccharide resulted in three saccharides coordinated in sites 1F, 3F and 6F (Fig 6B). In all cases, the Fuc moiety of the trisaccharide is recognized in the same way as with Me-α-l-Fuc. The oligosaccharide conformation is identical in all binding sites. In addition to interactions of the Fuc part with the protein, the trisaccharide in site 1F is further stabilized by the interaction of Gal-O4 with the Gly76 backbone and interaction of GlcNAc-N2 and GlcNAc-O3 with the Trp118 backbone. Analogously, in site 3F the saccharide interacts with the Gly171 and Trp214 backbone, in site 6F with the Gly315 and Trp355 backbone. The conformation of the trisaccharide is further stabilized by a water molecule bridging Gal-O4, Gal-O5 and GlcNAc-O3 to backbone of Trp118, Trp214 and Trp355, respectively. An additional water molecule was detected in sites 1F and 3F, bridging Gal-O3 with Gly76 and Gln77 (site 1F) and Gal-O3 with Gly171 (site 3F).

**Fig 7:**
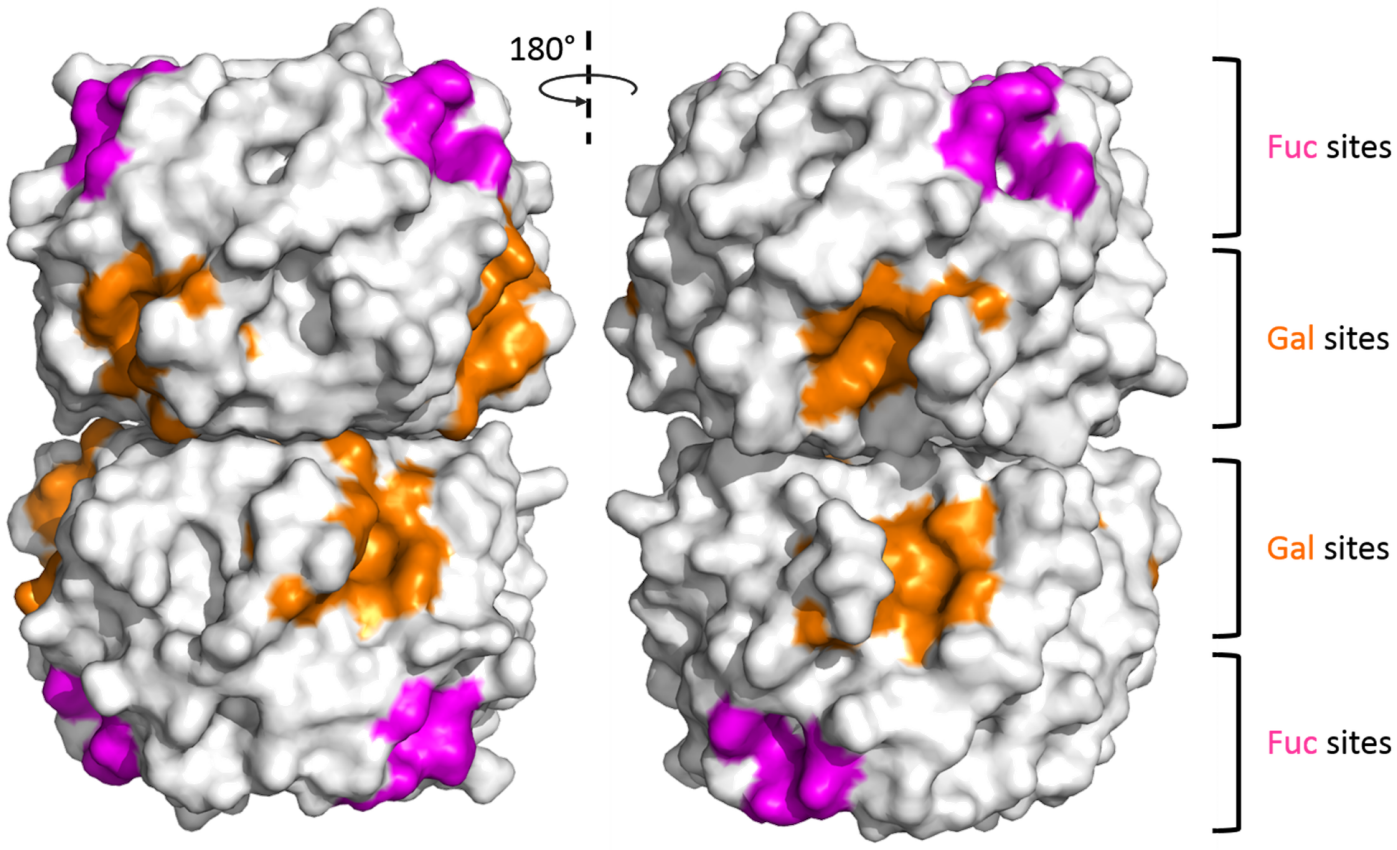
Localization of Fuc- and Gal-specific sites with respect to the PHL dimer. Residues closer than 4Å from the observed ligands are coloured.

The crystal structure of the PHL/d-Gal complex revealed three d-Gal monosaccharides coordinated in binding pockets other than the Fuc-binding sites. These Gal-binding sites are located in between blades in the opposite half to the N- and C- termini of the protein monomer (Fig 7). Similarly to Fuc-binding sites, potential Gal-binding sites are further reffered to as 1G (between blades W1 and W2), 2G and so on. The binding sites consist of several polar amino acids forming a complex hydrogen bond net with Gal oxygen atoms. For site 2G, Gal-O1, O2, O3, O4 and O5 atoms are coordinated by Arg91, Glu93, Gln155, Ser160 and Trp163 side chains and by the Gly161 main chain (Fig 6C). The Trp107 side chain stabilizes the hydrophobic surface of C1, C3 and C5 atoms of the coordinated Gal molecule through CH-π interactions. The saccharide is further stabilized by water molecules bridging the sugar hydroxyls to His105, Trp107, Gln119 and Ser160 side chains and the His159 main chain. The ligand at site 4G is coordinated in the same manner by Arg186, Glu188, Gln251 and Trp259 side chains, the Gly256 main chain and through a stacking interaction with a Trp202 side chain. Water bridges to Arg186 and Ser256 side chains and His255 main chain were also detected. In site 6G the hydrogen bonds with the Arg282, Glu284, Gln347 and Trp352 side chains are established, while the Trp298 side chain stabilizes the hydrophobic part. An additional water molecule forms a bridge to the Ser351 main chain. In all binding sites, only the beta anomer of d-Gal was identified. It is oriented in such a way, that only a Gal monomer could be bound. In sites 2G and 4G two conformations of Gal-C6 hydroxyl were identified as not being directly stabilized by the protein residues.

Based on sequence alignment of the repetitions (Fig 5C), only 5 Gal-binding sites contain the sugar binding motif. The conserved Trp residue is missing in sites 2G and 7G.

Interestingly, site 2F is occupied by the Met60 side chain of the symmetry-related molecule in all structures. For site 7F in PHL/ Me-α-l-Fuc and PHL/d-Gal complexes, electron density corresponding to another Met side chain was found (Fig 6A). This residue was identified as the initial Met1, since no other unassigned Met residue is present in the crystal. In addition, the electron density was also clear enough to assign Gln2 and Pro3 residues in the PHL/d-Gal complex. An overview of site occupancy of PHL protein by sugars is given in Table 4.

**Table 4:** Site occupancy of PHL-sugar complexes.

		PHL	PHL/Me-α-l-Fuc	PHL/BGH	PHL/d-Gal
Fucose-binding site	Site 1F			BGH	
	Site 2F	Met60*	Met60*	Met60*	Met60*
	Site 3F		Me-α-l-Fuc	BGH	
	Site 4F				
	Site 5F				
	Site 6F		Me-α-l-Fuc	BGH	
	Site 7F		Met1**		Met1**
Galactose-binding site	Site 1G				
	Site 2G				d-Gal
	Site 3G				
	Site 4G				d-Gal
	Site 5G				
	Site 6G				d-Gal
	Site 7G				

*Met60 from symmetry related PHL molecule

**The only Met available in the crystallization mixture is Met1 from identical or symmetry-related PHL molecule

### Biological activity of PHL

Several methods were used to prove the biological influence of PHL on human blood and insect haemolymph. Using human blood samples, its interaction with red blood cells (RBCs), effect on production of cytotoxic factors and serum antibacterial activity were determined. The potential influence of PHL on insects was tested on haemolymph: its antimicrobial and phenoloxidase activity were measured.

**Fig 8:**
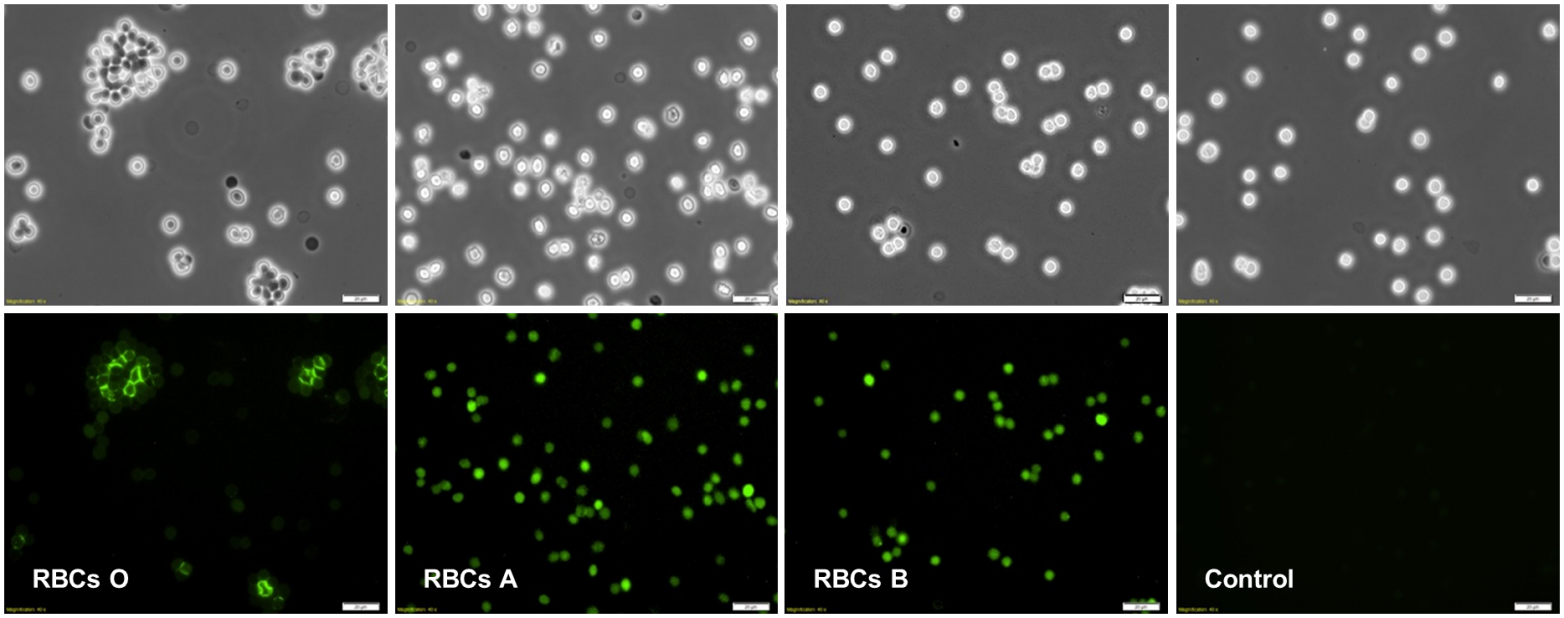
Fluorescence microscopy of interaction between PHL labeled with FITC and human red blood cells (RBCs). Visible light (upper) and fluorescent (lower) images of RBCs treated with PHL-FITC and control (RBCs O) without the lectin. RBCs were incubated with PHL-FITC at 17°C for 30 min, washed three times with PBS and observed under a microscope. PHL interacted with all types of RBCs but only RBCs O are visibly agglutinated.

The carbohydrate specificity of PHL lectin was observed through its interaction with the surface oligosaccharides of human RBCs under microscope. PHL displays considerable haemagglutination activity with RBCs of blood group O, but not with blood groups A and B. Experiments with FITC-labeled PHL under fluorescence microscope revealed that PHL was able to bind all RBC types, but only O RBCs were agglutinated (Fig 8). A set of biologically relevant mono- and oligosaccharides was used to determine their inhibition potency on haemagglutination by PHL. Interestingly, the best inhibitor proved to be the blood group B trisaccharide (its minimal inhibitory concentration (MIC) was 0.313 mM) followed by Me-α-l-Fuc (MIC 0.390 mM) that are 10 times and 8 times more effective than l-fucose (MIC 3.125 mM), respectively (Table 5). This indicates that blood group oligosaccharides are well recognized in their free form (terminal trisaccharides), while in bound form the least sterically demanding one (BGH) is preferred. Except for fucosylated sugars, no other carbohydrates inhibited the haemagglutination reaction at any of the concentrations tested (up to 250 mM).

**Table 5:** Haemagglutination inhibition assay with PHL using microscopy. Minimal inhibitory concentrations (MIC) of carbohydrate ligands and their potency towards l-fucose were determined.

Sugar	MIC [mM]	Potency
BGB trisaccharide	0.313	10
Me-α-l-Fuc	0.390	8
BGA trisaccharide	2.500	1.3
Fucα1-4GlcNAc	2.500	1.3
Fucα1-3GlcNAc	2.500	1.3
**l****-Fuc**	**3.125**	**1**
Me-β-l-Fuc	6.250	0.5
d-Man, d-Gal, d-Glc, d-GlcNAc, BGH trisaccharide	>250	<0.01

**Fig 9:**
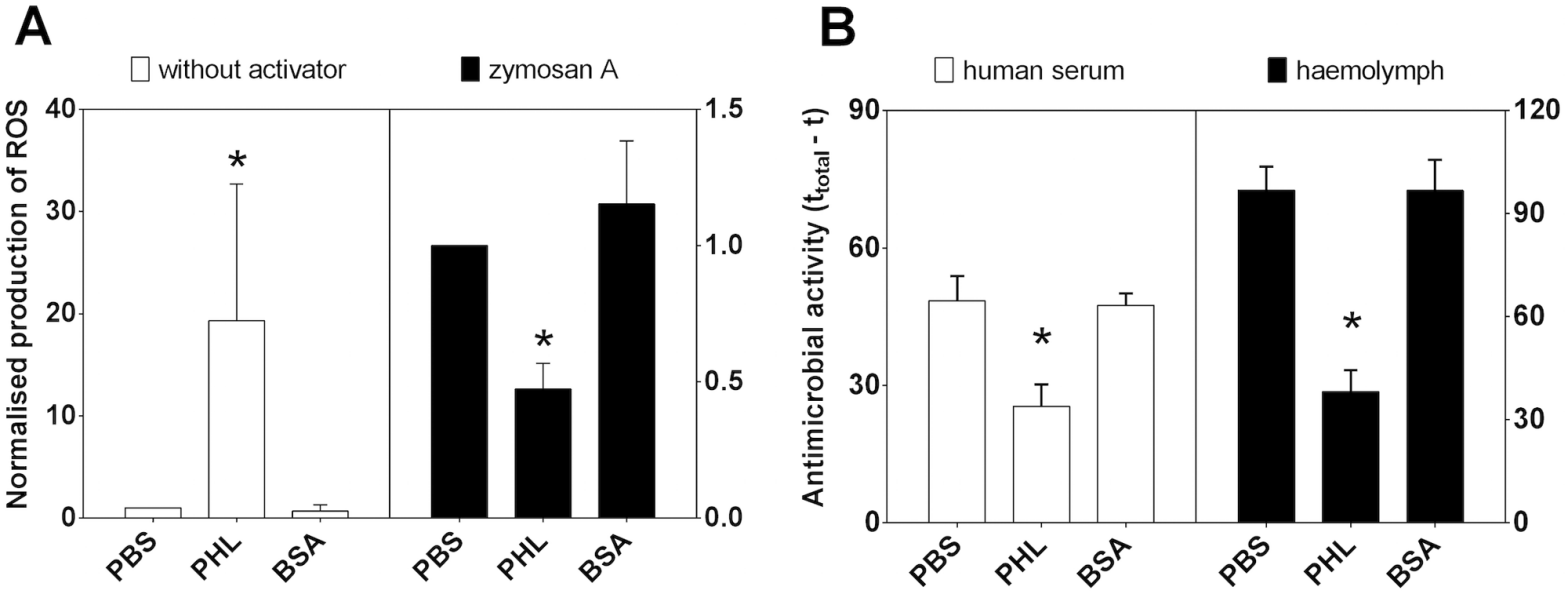
PHL effects production of reactive oxygen species (ROS) in human blood and impairs antimicrobial activity against *E*. *coli* K12. (A) Whole blood was treated with PHL (125 μg) or control protein BSA (125 μg), and the subsequent production of ROS was measured luminometrically at 37°C in the absence (without activator; white columns) or presence of the activator zymosan A (black columns). The presented data show the integral of ROS production in blood with PHL or BSA normalised to the integral of the blood reaction with PBS ± SD. (B) Human serum (white columns) and *G*. *mellonella* haemolymph (black columns) were treated with PBS, BSA (320 μg) or PHL (320 μg) and then mixed with bioluminescent bacteria *E*. *coli* K12. The measured luminescence signal is directly proportional to the number of live bacteria. Antimicrobial effect is expressed as the difference between the total time of measurement (t_total_; 120 min for human serum and 90 min for haemolymph) and the time (t) when the luminescence signal dropped below 1000 or 5000 CPS for human serum and haemolymph, respectively. Results from three independent measurements are shown ± SD. * indicates significant difference (p < 0.05; Wilcoxon test).

PHL was shown to bind to host cells, but interestingly we also observed a modulation of the host immune response when the lectin was present. Reactive oxygen species (ROS) are highly effective oxidants which are produced by immune cells after pathogen recognition and are responsible for the primary antimicrobial response. The level of ROS production was very low in whole human blood A/B/O in the absence of immune activators both in the blood mixed with PBS (integral of bioluminescence 45.7±11.2 x 10^3^ counts per second, CPS, during 2 hours of reaction) and control protein BSA (26.9±16.9 x 10^3^ CPS*min). In contrast, the constitutive production of reactive oxidants was significantly increased in blood which was pre-treated with PHL (926.2±650.4 x 10^3^ CPS*min), confirming the interaction of the lectin with immune cells and its recognition by the immune system (Fig 9A). Interestingly, in the blood challenged with the activator zymosan A, the production of ROS was significantly lower after pre-treatment with PHL (2.8±1.0 x 10^6^ CPS*min) compared to PBS and BSA (6.5±3.6 x 10^6^ and 5.3±1.3 x 10^6^ CPS*min, respectively). Mixing blood with PBS and BSA neither affected the oxidative burst in phagocytes positively nor negatively. It is worth noting that the level of ROS produced in blood with the activator was more than 140-fold higher than their constitutive level (without the activator); this is not apparent from the normalised results shown at Fig 9A. In the presented results, the integral of ROS production was normalized to the PBS control for each particular sample of blood to exclude blood donor variability. The effect of PHL was apparent both at room temperature and 37°C; the temperature influenced only course of the reaction which was faster in human optimum at 37°C.

Inhibition of antimicrobial response was also observed upon the challenge of immune effectors with live bacteria. An antimicrobial assay using the Gram-negative bacteria *E*. *coli* K12 found a weaker antimicrobial activity in human serum and insect haemolymph treated with PHL (Fig 9B). The viability of the bacteria used is not influenced in the absence of human serum and haemolymph, but decreases in the presence of body fluids containing antimicrobial factors. Unlike PBS or BSA that are not able to block the effect of antimicrobial factors present in body fluids, PHL interacts both with human serum and haemolymph, resulting in a delay of the observed antimicrobial effect. PHL-dependent inhibition of antimicrobial activity was also observed in whole human blood, yet the measured luminescence and variability of results were negatively influenced by the presence of erythrocytes and absorption of haemoglobin, therefore these data are not presented.

**Fig 10:**
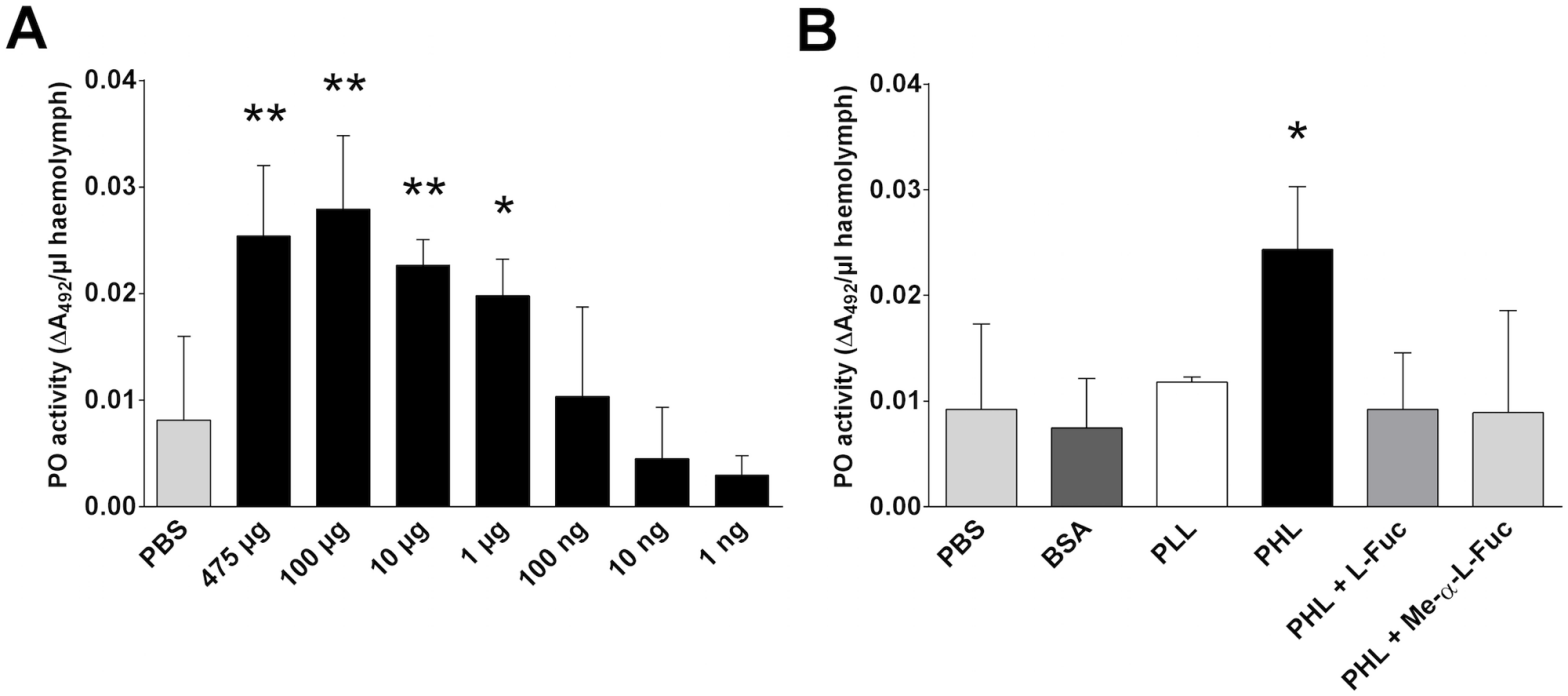
Phenoloxidase (PO) activity in haemolymph of *G*. *mellonella*. (A) Haemolymph samples were incubated with PBS or different amounts of PHL and the melanisation catalysed by PO was measured at 492 nm after mixing with 3,4-dihydroxy-dl-phenylalanine substrate solution. (B) Haemolymph was incubated with PBS, BSA (475 μg), PLL (475 μg) or PHL (475 μg; active or inhibited by 0.2M l-Fuc and Me-α-l-Fuc) and subsequently mixed with the substrate 3,4-dihydroxy-dl-phenylalanine. Products of melanisation catalysed by PO were measured at 492 nm. Data presented as means ± SD; * indicates significant difference p < 0.05, ** p < 0.01 (Dunnett's and Tukey’s test, respectively).

Although, the ROS production is still disputable in some insect species [25,26], even they possess immune mechanisms that are able to respond quickly to pathogen recognition. The phenoloxidase (PO) cascade is well described in insects, activated upon the detection of pathogen structures and accompanied by the production of cytotoxic factors [27]. The effect of PHL on PO activity was examined in *G*. *mellonella* haemolymph where the PO activity is manifested in rapid melanisation. A significant increase in melanisation was observed in haemolymph treated with micrograms of PHL (Fig 10A). This activation was inhibited in the presence of both 0.2M l-Fuc and 0.2M Me-α-l-Fuc (Fig 10B). Unlike PHL, the high doses of homologous protein PLL and control protein BSA did not have any effect on PO activity.

## Discussion

A functionally unique lectin PHL from the insect and human pathogen *Photorhabdus asymbiotica* was identified as a homologue of a recently published lectin from the strictly entomopathogenic bacterium *Photorhabdus luminescens* [21]. Individual steps of *P*. *asymbiotica* human infection have not been determined, however lectins produced by this pathogen can play an important role in its pathogenicity and/or the process of infection. The aim of this work was to prepare a novel PHL lectin and describe its structure-functional properties. Its biological relevance was tested on human blood and insect haemolymph, with an emphasis on early immune response and lectin interaction with circulating cells. PHL, a homo-dimer lectin (~ 80.4 kDa) with subunits linked by a disulfide bridge, was characterized as a dual-specific l-fucose/d-galactose binding protein with an unusually high number of potential binding sites. The results of biological activity assays suggest that PHL significantly affects host defense mechanisms, specifically antimicrobial effectors such as reactive oxygen species and their production.

The structure of PHL was solved using the structure of the lectin PLL from *Photorhabdus luminescens* [21]. Similarly to PLL, the PHL monomer consists of a seven-bladed β-propeller that has not been observed in any other lectin [21]. The seven-bladed propeller of PVL from *Psathyrella velutina* differs in the formation of its blades, the insertion of its N-terminus into the central cavity, and also in its larger propeller diameter. Propellers of the AAL lectin family are designed in a similar way to PHL, but contain only six blades. In contrast to tetrameric PLL, PHL exists as a dimer stabilized through a disulfide bridge. This is remarkable, as the protein is produced in an *E*. *coli* strain not expected to support disulfide formation, however this uncommon feature was already reported for PLL [21]. Unlike all lectins studied so far, PHL is unique in the presence of two sets of binding sites in one domain. So-called superlectins consist of two different domains, each with a different specificity (e.g. BC2L-C from *Burkholderia cenocepacia* or CRLL lectin from *Cycas revoluta*) [28,29]. Also, the ABL from *Agaricus bisporus*, the SRL from *Sclerotium rolfsii* or XCL from *Xerocomus chrysenteron* possess the ability to bind two different saccharides within one unit [30,31]. However, two sets of well-defined binding sites specific for two unrelated saccharides has never been reported before. Hence this is a unique bangle lectin arrangement. Remarkably, due to architecture of PHL, all potential binding sites are exposed at its surface and therefore accessible to ligands in solution.

**Fig 11:**
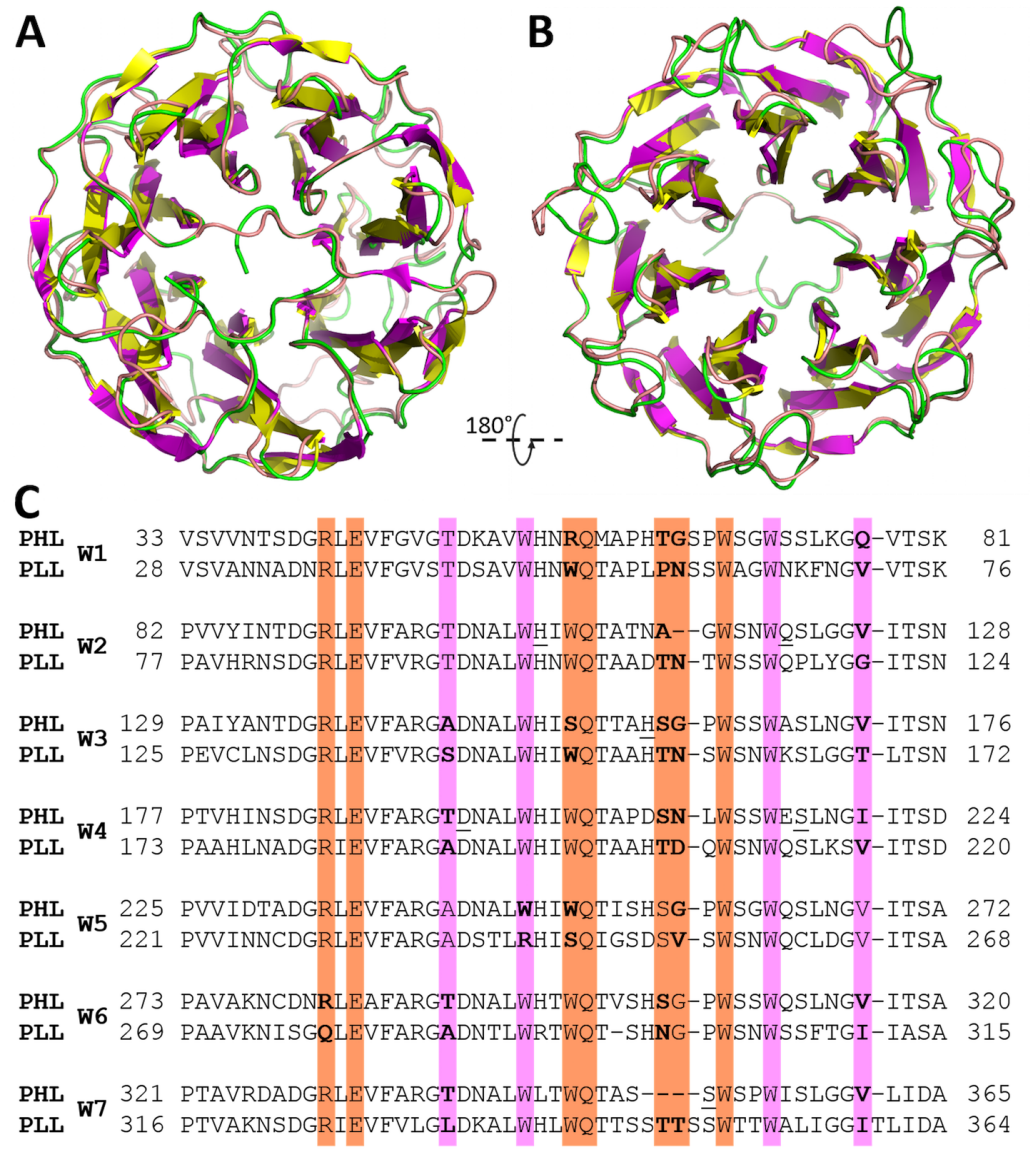
Comparison of PHL/Me-α-l-Fuc and PLL/l-Fuc (PDB ID: 5C9P) structure. Top (A) and bottom (B) view of PHL (yellow/green) and PLL (magenta/pink) monomer. Differences are mainly visible in loop regions. (C) Sequence alignment of PHL/PLL repetitions. Residues responsible for l-Fuc or d-Gal binding are highlighted with a magenta and orange background, respectively. Different residues in the binding sites shown in bold.

The PHL fucose-binding site exhibits a high similarity to the recently characterized binding site of homologous PLL [21]. It shares the characteristic employment of two Trp residues for ligand binding via CH-π interactions rather than creating a complex structure of H-bonds, as is common for fucose-specific AAL family lectins [32–35]. In the structure of the PLL complex with l-Fuc, the saccharide was observed in three binding sites. Similarly, three fucose sites were occupied in the PHL/BGH complex, however, the distribution of confirmed binding sites is not equal, only two of them sharing the same position and the third one being different. Moreover, the crucial amino acids in the fucose sites of PHL are more conserved than in PLL (Fig 11), where at least one site lacks a conserved Trp residue.

The uniqueness of PHL resides in the presence of the second set of binding sites within the same monomer. These sites form a ring around the PHL monomer below the fucose-binding sites (Fig 7) and, in contrast to them, prefer d-galactose. Galactose-binding sites exhibit a common binding scheme, with the hydrophobic part of the saccharide being stabilized by the Trp residue, while the rest of the binding pocket coordinates the saccharide hydroxyl groups. Even though PHL exhibits submillimolar affinity towards d-Gal, it is unlikely to be its natural ligand. Lectins are mostly considered to be oligosaccharide-recognizing proteins, especially if the saccharides are linked to a surface (such as to the cell, another protein or intercellular matrix). In the PHL/d-Gal complex, the d-Gal anomeric oxygen is oriented towards the bottom of the binding pocket, so that recognition of the more complex saccharide is sterically hindered. This corresponds well with the inability of PHL to recognize a d-Gal-modified surface in SPR measurements. Hence, we can assume that the natural ligand is different from d-Gal, e.g. d-Man, which is utilized for PHL purification and exhibits low overall affinity.

Glycan microarray experiments demonstrated that the binding of PHL is highly selective for fucosylated compounds out of more than 600 tested glycans, and therefore PHL was defined as a fucose-specific lectin. The highest relative binding was observed with α-l-fucoside. PHL displayed significant preferences for short glycans, as is demonstrated by its preference for Fucα1-4GlcNAc and Fucα1-3GlcNAc disaccharides over complex fucosylated saccharides, such as Lewis antigens or blood group ABH determinants. Unexpectedly, among the highest responding epitopes unusual *O*-methylated saccharides were found– 3,6-*O*-Me_2_-Glcβ1–4(2,3*-O*-Me_2_)Rhaα-*O*-phenol (the terminal disaccharide from *Mycobacterium leprae* glycolipid I) and monosaccharide 3,6-*O*-Me_2_-Glc*p*β [22,23]. It is interesting to know that *O*-methyl glycans are frequently present in some species of bacteria, fungi, algae, plants, worms (e.g. nematodes) and molluscs, but not in mammals [36,37]. The results published in [37] also suggest that *O*-methylated glycans constitute a conserved target of the fungal and animal innate immune system. Therefore, the specificity of PHL to *O*-methylated glycans can point to its role in interactions with both nematodes and insects. It also nicely corresponds with specificity of PLL [21].

The ITC measurement revealed a different behavior in solution, where more complex oligosaccharides such as blood group A/B trisaccharides were determined to have a higher affinity, comparable to the above-mentioned disaccharides. Blood group B trisaccharide and d-galactose turned out to be more strongly bound ligands than l-fucose. This apparent discrepancy follows from differences between the surface assay and solution assay. This is further supported by the fact that d-galactose can probably be only bound in free form, as was demonstrated by the X-ray structure of the PHL/d-Gal complex.

In comparison with PLL, PHL has a higher affinity to both l-Fuc and Me-α-l-Fuc. During ITC measurements, PHL interacted with both saccharides with an approx. 4-fold higher affinity. IC_50_ based on SPR inhibition experiments found a 15-fold better inhibition effect of l-Fuc for PHL than for PLL and even a 42-fold stronger effect with Me-α-l-Fuc. There are also differences in their haemagglutination assays. PHL binds all types of RBCs, but agglutination was observed only for O RBCs. This might be caused by several factors including PHL binding site non-equivalency, partial sterical hindrance of cell-bound saccharide or local interference with other epitopes on the cell [38]. However, it is interesting that homologous PLL agglutinates only A RBCs, showing different overall behaviour to RBCs [21].

*P*. *asymbiotica* is known as a facultative intracellular pathogen which is engulfed by human macrophages and insect haemocytes, but is able to avoid destruction and later disseminate from phagocytic cells [3]. Therefore, *P*. *asymbiotica* must possess efficient defense mechanisms that enable survival of the detrimental effects of antimicrobial compounds constitutively present or induced in the host organism upon its recognition. We focused on early immune response and its effectors, which are responsible for the primary response to pathogens such as *P*. *asymbiotica*. PHL interacts with host cells and was also shown to interfere with the production of ROS in human blood. The observed increase in constitutive ROS level in all blood types, including A and B that are not agglutinated by PHL, suggests the possible recognition of this lectin by the immune system. *P*. *asymbiotica* as well as *P*. *luminescens* and *P*. *temperata* are able to kill insect haemocytes and vertebrate macrophages [3, 39] and higher concentrations of ROS can certainly contribute to this by causing oxidative damage in host tissues. However, it is worth noting that the apoptosis of host cells occurs later, and the level of ROS produced after immune activation was significantly higher than the amount of oxidants produced in reaction to PHL itself. Overall, in our experiments we did not observe any detrimental effect of PHL on host cells, which could be attributed to increased ROS concentration. Moreover, PHL was able to impair the ability of human blood cells to produce ROS after immune activation by zymosan A, which indicates the major role of PHL is to help in overcoming host defenses instead of evading their activation. The observed inhibitory effect of PHL on oxidant production could further contribute to the reported survival of *P*. *asymbiotica* in mammals [3], which is essential for its clinical manifestation.

The PHL-dependent inhibition of antimicrobial response was further confirmed using live bacteria *E*. *coli*; the lectin was able to impair the antimicrobial effect of both whole blood and haemolymph and thus provide bacteria with more time for their growth, which is accompanied by the reported depletion of host nutritional resources [5]. Surprisingly, we also observed inhibitory activity in human serum, suggesting that the PHL-mediated immune suppression is not only limited to the inhibition of cellular immunity. Cell-free body fluids of vertebrates and invertebrates contains potent antimicrobials, such as antimicrobial peptides, complement proteins or complement-like molecules with bacteriostatic or bactericidal effect [40–42]. In particular, the complement cascade is known for its activation by lectins. The mechanism of PHL interaction with the humoral immunity of the host is not known, but their competition in binding to bacterial surfaces can be assumed.

An important fact, which must be taken into account when considering the ecological relevance of PHL, is the amount of the lectin produced by *P*. *asymbiotica* during infection. To inhibit the ROS production in human blood and weaken the antimicrobial activity of human serum, the dose of 100 μg was needed in our experiments (S1 and S2 Figs). It is supposed that PHL is produced in significantly lower amounts within natural conditions, however, the activity of PHL during infection could be promoted by cooperation with other factors produced by bacteria and possibly even their nematode vector which might result in increased effectivity of lower lectin concentrations.

Taken together, PHL might act as inhibitor of antimicrobial response, although it seems it is recognized by host defenses. Interestingly, more pronounced effect of PHL was observed on phenoloxidase activity in *G*. *mellonella* haemolymph, in which it induced melanisation, the reaction activated by pathogen-associated molecular patterns, aberrant tissues or artificial particles and mediated by enzyme phenoloxidase [27,43]. It is of note that two proteins, BSA and PLL, are not recognized in the same way and do not activate haemolymph melanisation. Similar to PHL, the metalloprotease PrtS from *P*. *luminescens* TTO1 described previously was associated with the induction of melanisation in insects [44]. Although the precise function of PrtS in the infection process was not specified, a role in the depletion of melanisation response is assumed. The fact that PHL activates constitutive immune mechanisms and at same time is able to restrict antimicrobial activity after immune challenge suggests a possible role of this lectin in depleting the host immune response accompanied by limiting its harmful effects on *P*. *asymbiotica* itself. The characterization of the precise mechanism of the PHL-mediated inhibition of antimicrobial response is of interest for further studies, since it could reveal novel approaches to control *P*. *asymbiotica* and related pathogens.

In summary, the lectin from *Photorhabdus asymbiotica* was revealed as the first example of a protein with up to twelve potential binding sites in one domain with dual specificity. PHL recognizes fucose and its derivates with micromolar affinity and also an unusual terminal *O*-tetramethylated disaccharide from *Mycobacterium leprae*. Fucosylated carbohydrates are widely found on human cells, *O*-methylated sugars on the cells of bacteria, fungi, algae, plants, worms (e.g. nematodes) and molluscs. In addition, interaction with human blood cells and haemocytes revealed not only binding to cell surfaces, but also modulation of the immune response. Taken together with the observed inhibition of antimicrobial activity in human serum, our results indicate that PHL plays an important role in insect and human infections.

## Materials and methods

### Materials

Methyl-α-l-fucopyranoside, methyl-β-l-fucopyranoside, d-glucose and d-galactose were purchased from Carbosynth, Compton, United Kingdom; blood group A/B/O trisaccharides, and Fucα1-GlcNAc and Fucα1-GlcNAc were purchased from Carbohydrate Synthesis, Oxford, United Kingdom; l-fucose was purchased from Applichem, Darmstadt, Germany. *N*-acetyl-d-glucosamine, d-mannose, d-mannose-agarose, biotin and streptavidin, bovine serum albumin, 3,4-dihydroxy-dl-phenylalanine, zymosan A from *Saccharomyces cerevisiae* and luminol were purchased from Sigma-Aldrich, St Louis, USA. Biotinylated saccharides (biotinylated α/β-d-mannoside, α/β-d-galactoside and α-l-fucoside) were purchased from Synthaur LLC, Moscow, Russia. Fluorescein isothiocyanate (FITC) and DyLight 488 were purchased from ThermoScientific, Rockford, USA. Basic chemicals were purchased from Sigma-Aldrich, St Louis, USA; Duchefa, Haarlem, Netherlands; ForMedium, Hunstanton, United Kingdom and Applichem, Darmstadt, Germany.

### Gene cloning

The sequence of *P*. *asymbiotica* lectin PHL was identified in the genome of ATCC43949 strain [7] with the NCBI Blast tool using the sequence of PLL lectin from *P*. *luminescens* (UniProt ID: Q7N8J0) as a probe. The nucleotide sequence coding for the peptide sequence of PHL was synthetized by Life Technologies with optimization for expression in *E*. *coli* and flanking with the *Nde*I and *Hind*III restriction endonucleases. The DNA sequence of the whole gene was inserted into the cloning site of expression vector pET25b (Novagen) using *Nde*I and *Hind*III restriction sites, resulting in the plasmid pET25b_*phl*. The construct does not introduce any tags at the C- and N-terminus of the recombinant protein. The vector of interest was transformed into *E*. *coli* XL1 using ampicillin for plasmid propagation. For the protein production, the vector pET25b_*phl* was transformed into *E*. *coli* Tuner (DE3) cells (Novagen). The sequence of the plasmid pET25b_*phl* and its presence in transformed *E*. *coli* cells were confirmed by restriction cleavage of the re-isolated plasmid and its sequencing.

### Protein expression and purification

*E*. *coli* Tuner (DE3)/pET25b_*phl* cells were grown in standard LB broth low-salt medium (ForMedium, UK) containing 100 μM ampicillin at 37°C until the OD_600_ reached ~ 0.5. After induction with 0.2 mM isopropyl ß-d-1-thiogalactopyranoside (ForMedium, UK), cells were cultured for an additional 20 hours at 18°C, harvested by centrifugation at 12,000 g for 10 min and resuspended in buffer A (300 mM NaCl, 20 mM Tris/HCl, pH 7.5). Harvested cells were stored at -20°C prior to protein purification.

Cells were disrupted by sonication (VCX 500, Sonics & Materials, Inc., USA) and the soluble fraction was collected by centrifugation at 21,000 g at 4°C for 1 hour and filtrated through a 0.45 μm pore size filter (Carl Roth, Germany). Recombinant protein PHL was purified by affinity chromatography on mannose-agarose resin (Sigma-Aldrich, USA) equilibrated with buffer A using an ÄKTA FPLC system (GE Healthcare, UK). The protein was eluted isocratically. Protein purity was assessed by SDS-PAGE (12% gel) stained with Coomassie Brilliant Blue R-250/G-250 (Sigma-Aldrich, USA). The fractions containing pure PHL were extensively dialyzed against an appropriate buffer and used for further studies. If desired, PHL was concentrated using an ultrafiltration unit with a 10-kDa cut-off membrane (Vivaspin 20, Sartorius, Germany).

### Glycan array

Purified PHL lectin samples were labelled with DyLight 488 NHS Ester (Thermo Scientific) according to the manufacturer’s instructions and dialysed against PBS buffer (137 mM NaCl, 2.7 mM KCl, 8 mM Na_2_HPO_4_, 1.47 mM KH_2_PO_4_, pH 7.4). The labelled protein was used for glycan array screening following the manufacturer’s standard procedure (Semiotik, Moscow, Russia). To determine the lectin specificity, the screening of the printed glycan microarray chip (slide number 10085636, with ~400 mammalian glycans and ~200 bacterial polysaccharides, all in 6 replicates) was performed with a PHL concentration of 200 μg/ml in PBS buffer. The relative binding of PHL was calculated as an average fluorescence from six replicates of each saccharide present in the array.

### Surface plasmon resonance

SPR experiments were performed in a BIAcore T200 instrument (GE Healthcare) at 25°C. The carboxymethyldextran surface of a CM5 (GE Healthcare, UK) sensor chip was activated with *N*-ethyl-*N*-(3-dimethylaminopropyl)carbodiimide/*N*-hydroxysuccinimide solution according to the manufacturer’s standard protocol using HBS buffer (10 mM HEPES, 150 mM NaCl, 0.05% Tween 20, pH 7.5). Streptavidin was immobilized into all four channels to a final response of 6,300–7,500 RU. Subsequently, the sensor surface was blocked with 1 M ethanolamine. Biotinylated α/β-d-mannoside, α/β-d-galactoside and α-l-fucoside were injected into three measuring channels (final response ~ 140 RU) and pure biotin in the blank channel at a flow rate of 5 μl/min. In the experimental setup, measurements were carried out simultaneously in all four measuring channels using buffer A (300 mM NaCl, 20 mM Tris/HCl, pH 7.5) supplemented with 0.05% Tween20 at a flow rate of 20 μl/min. The interaction of PHL with immobilized sugars was measured over the concentration range 200–0.16 μg/ml.

SPR inhibition experiments were performed using the same conditions described above. The PHL lectin (0.25 μM final concentration) was mixed with various concentrations of inhibitors (1M – 0.01 mM) and injected onto the sensor chip. Pure PHL lectin was used as a control (0% inhibition). The response of the lectin bound to the sugar surface at equilibrium was plotted against the concentration of inhibitor in order to determine IC_50_.

### Isothermal titration calorimetry (ITC)

ITC experiments were performed using an ITC200 calorimeter (Malvern, England). Experiments were performed at 25°C in buffer A (300 mM NaCl, 20 mM Tris/HCl, pH 7.5). Carbohydrate ligands were dissolved in the same buffer. Protein in the cell (50 μM) was titrated by consecutive addition (2 μl) of the ligand in the syringe (1.5–50 mM) while stirring at 1000 rpm. Control experiments performed with injections of buffer in the protein solution yielded insignificant signals. Integrated heat effects were analyzed by nonlinear regression using a single-site binding model in Origin 7 (Microcal) [45]. The experimental data fitted to a theoretical titration curve brought up stoichiometry (*n*), association constant K_a_, and the enthalpy of binding ΔH. The other thermodynamic parameters such as free energy (ΔG) and enthalpy (ΔS) were calculated from the equation ΔG = ΔH-*T*ΔS = -*RT*lnK_a_, where *T* is the absolute temperature and *R* is the molar gas constant (8.314 J.mol^-1^.K^-1^). At least two independent titrations were carried out for each tested ligand.

### Analytical ultracentrifugation (AUC)

AUC experiments were performed using a ProteomeLab XL-A analytical ultracentrifuge (Beckman Coulter, California, USA) equipped with an An-60 Ti rotor. Before analysis, purified PHL was brought into the experimental buffer (20 mM Tris/HCl, 100 mM NaCl, pH 7.5) by dialysis and the dialysate was used as an optical reference.

Sedimentation velocity experiments were conducted in a standard double-sector centerpiece cell loaded with 420 μl of protein sample (0.05–0.21 mg/ml) and 430 μl of reference solution. Data were collected using absorbance optics at 20°C at a rotor speed of 42,000 rpm. Scans were performed at 280 nm at 6-min intervals and 0.003 cm spatial resolution in continuous scan mode. The partial specific volume of protein together with solvent density and viscosity were calculated from the amino acid sequence and buffer composition, respectively, using the software Sednterp (http://bitcwiki.sr.unh.edu). The sedimentation profiles were analyzed with the program Sedfit 14.3 [46]. A continuous size-distribution model for non-interacting discrete species was used to provide a distribution of apparent sedimentation coefficients.

Sedimentation equilibrium experiments were performed at 20°C in a six-channel centerpiece cell loaded with 110 μl of PHL (0.02, 0.05 and 0.09 mg/ml) and 120 μl of reference solution. The sample was gradually spun at rotor speeds of 9,500 rpm, 11,400 rpm, and 20,000 rpm, respectively. After equilibrium was achieved, data were collected at 280 nm by averaging 20 replicates with 0.001 cm spatial resolution in step mode. Data from the multi-speed experiment were globally analyzed with SEDPHAT 10.58 [47] using a non-interacting discrete species model with mass conservation constraints.

### Crystallization and data collection

The PHL protein was concentrated to 13 mg/ml using an ultrafiltration unit with a 10-kDa cut-off membrane (Vivaspin 20, Sartorius, Germany). Initial crystallization conditions were screened with the commercial screening kits Classic, Classic II, Classics Lite, PACT (Qiagen, Hilden, Germany), and Structure I+II suites (Molecular Dimension, UK) using the Mosquito crystallization robot (TTP LabTech, UK). Using the sitting drop vapour diffusion method, 0.2 μl drops of protein/precipitant solution were spotted on the crystallization plate and incubated at 17°C. PHL formed crystals within two weeks under several suitable conditions. After optimization, the final crystals were obtained under the following conditions: 4 μl sitting drop, protein solution mixed with precipitant (3.7–4.3 M NaCl, 100 mM HEPES pH 7.5) in ratios 1:1, 3:5, 1:3 and 1:7. The drops were set against 0.5–1 ml of the same equilibration precipitant. To determine the PHL structure complexed with ligands, the PHL was co-crystallized with 3 mM methyl-α-l-fucoside or the crystals of PHL were soaked in a 4 mM solution of BGH trisaccharide for 1.5 hour or 200 mM solution of d-galactose for 70 minutes, respectively. The crystals were cryo-protected using 40% PEG 400 and frozen in liquid nitrogen.

The diffraction data of free PHL were collected with an in-house Rigaku HighFlux HomeLab (Rigaku, Tokyo, Japan) robotized macromolecular diffraction system with ACTOR sample changer at the Cu-Kα wavelength. The diffraction data of PHL complexed with saccharides were collected at the BESSY II electron storage ring (Berlin-Adlershof, Germany) [48].

### Structure determination

Images were processed using XDSAPP [49] and converted to structure factors using the program package CCP4 v.6.5 [50], with 5% of the data reserved for R_free_ calculation. The initial structure of PHL was solved using the molecular replacement method with a homology model based on the structure of PLL [21] generated by a Phyre2 „one to one” approach [51]. The structures of PHL complexes were solved by molecular replacement with MOLREP [52] using the monomeric coordinates of the initial PHL structure. Refinement of the molecule was performed using REFMAC5 [53] alternated with manual model building in Coot v.0.8 [54]. Sugar residues and other compounds that were present were placed manually using Coot. Water molecules were added by Coot and checked manually. The addition of alternative conformations, where necessary, resulted in final structures that were validated using the ADIT (http://rcsb.org) and MolProbity [55]; http://molprobity.biochem.duke.edu] validation servers and were deposited in the PDB as entries 5MXE, 5MXF, 5MXG, 5MXH. Molecular drawings were prepared using Pymol (Schrödinger, Inc.).

### Haemagglutination

Red blood cells (RBCs) were washed four times with PBS buffer (137 mM NaCl, 2.7 mM KCl, 8 mM Na_2_HPO_4_, 1.47 mM KH_2_PO_4_, pH 7.4), diluted to 50% with PBS with 0.005% (w/w) sodium azide and treated with 0.1% papain. Haemagglutination assay with the prepared RBCs was performed using microscopy [56]. PHL (2 mg/ml) in PBS buffer and 10% RBCs were gently mixed in a 1:1 (v/v) ratio. The reaction mixture was incubated for 10 minutes and subsequently observed on microscope slides using a Levenhuk 2L NG microscope with a Levenhuk D2L digital camera (Levenhuk, USA).

A haemagglutination inhibition assay was performed to determine the specificity and semi-quantitative affinity of PHL interaction with the saccharides. A wide range of monosaccharides at concentrations from 50 mM to 0.5 mM were used for determining the lowest inhibiting concentration. A 20 μl mixture composed of 5 μl PHL (2mg/ml), 5 μl saccharide, and 10 μl 10% RBCs was prepared (all components of reaction were in PBS buffer). After 10 minutes, the reaction was observed using a Levenhuk microscope.

### Analysis of protein-saccharide interaction using fluorescence microscopy

The PHL protein was labelled with FITC according to the manufacturer’s instructions and dialysed against PBS buffer. 1 μl of labelled PHL protein (2 mg/ml) was incubated with 100 μl of cells (RBCs and haemocytes). As a control individual types of RBCs were used and mixed with 1 μl FITC prepared for labelling. The mixtures were incubated at 17°C for 30 minutes, the cells washed three times with PBS buffer and observed under a fluorescence microscope (OLYMPUS IX81 Microscope IX81F-3 with IX2-UCB-2 Controller and X-Cite 120PC Q; Olympus and Excelitas Technologies, Japan, resp. USA).

### Preparation of human serum for bioassays

Samples of human blood were collected from healthy donors to tubes without anticoagulant and anonymized prior to experiments. The blood was allowed to clot for at least 10 min, centrifuged at 2,000 g, 10 min to remove the clot and the collected serum was used immediately for subsequent analysis. The serum was analysed within two hours after blood collection in all experiments.

### Haemolymph collection

Larvae of the greater wax moth, *Galleria mellonella*, were reared on an artificial diet [57] at 30 ± 1°C in constant darkness. Haemolymph was collected from the seventh instar larvae by cutting a proleg and pooling the haemolymph into a tube containing phenylthiourea (1 mg/ml in PBS; haemolymph mixed with phenylthiourea in the ratio 9:1 [v/v]) or used directly for the determination of phenoloxidase activity.

### Oxidative burst in human blood

ROS production was measured according to previously published work [58] in whole human blood within 15 min of sampling. The fresh human blood (2 μl diluted 40x in HBSS; 0.137 M NaCl, 5.4 mM KCl, 0.44 mM KH_2_PO_4_, 0.25 mM Na_2_HPO_4_, 4.2 mM NaHCO_3_, 1.0 mM MgSO_4_, 1.3 mM CaCl_2_, 5.55 mM glucose; pH 7.4) was mixed with 25 μl of PHL (125 μg in PBS for all experiments except dose-dependence assay where different dilutions of PHL were used), BSA (125 μg in PBS) or PBS (pH 7.4) and incubated for 10 min at 37°C or room temperature. After incubation, 25 μl of the luminophore (10 mM luminol) and 25 μl of activator zymosan A (2.5 mg/ml in HBSS) was added to activate ROS production in phagocytes. To detect the constitutive production of ROS, zymosan was replaced with the same volume of HBSS. Luminescence was recorded with a Chameleon V luminometer (Hidex, Finland) for two hours at 37°C or room temperature, and the integrals of reactions were compared. Results from experimental treatments (incubation of blood with PHL and BSA) were normalised to the integral of the oxidative response in the respective blood sample with PBS.

### Antimicrobial activity

The antimicrobial activity of human sera and insect haemolymph was measured luminometrically using the bioluminescent Gram-negative bacteria *Escherichia coli* K12 [59]. We have modified previously published protocol to measure the effect of PHL [60]. Briefly, 16 μl of serum or haemolymph was incubated with 64 μl PHL (320 μg in PBS for all experiments except dose-dependence assay where different dilutions of PHL were used), BSA (320 μg in PBS) or PBS (treatment control) at room temperature for 10 min. After treatment, 120 μl of bacteria working solution containing 100,000 *E*. *coli* K12 cells in PBS (pH 7.0) was added to the reaction well and the luminescence signal was recorded with a Chameleon V luminometer (Hidex, Finland) in counts per second (CPS). Reaction wells with PHL, BSA or PBS in an 80 μl volume of the same concentrations and pH as above were measured with *E*. *coli* K12 in each experiment as viability controls, not affected by human serum nor haemolymph. The luminescence produced by *E*. *coli* K12 corresponds to the bacterial viability. The antimicrobial effect of human sera and haemolymph was determined as time elapsed until the luminescence signal decreased under 1000 or 5000 CPS, respectively. The determined time was subtracted from the total time of the assay, 90 min for human sera and 120 min for haemolymph, so that higher values would correspond to higher antimicrobial activity.

### Phenoloxidase activity in insect haemolymph

PO activity was measured according to previously published studies [61,62]. Haemolymph of *G*. *mellonella* (5 μl) was collected directly to 95 μl of PHL (475 μg in PBS for all experiments except dose-dependence assay where different dilutions of PHL were used), PLL (475 μg in PBS), BSA (475 μg in PBS) or PBS (pH 7.4) and incubated for 10 min at room temperature. After incubation, 40 μl of reaction mixture was transported to the microplate well and 160 μl of 3,4-dihydroxy-dl-phenylalanine (3 mg/ml in PBS) was added as substrate. The reaction was allowed to proceed for 30 min, and absorbance was measured in 2-min intervals at 492 nm with a Sunrise reader (Tecan, Switzerland). PO activity was expressed as linear increase in absorbance per minute. For the inhibition assay, PHL was pre-incubated with 0.2M l-Fuc or Me-α-l-Fuc for 10 min at room temperature prior to mixing with haemolymph.

### Statistical analysis

Data were analysed in Statistica 12 (StatSoft, USA). To test the effect of protein treatment on antimicrobial activity and oxidative burst, the results from related samples were compared using the Wilcoxon test. Normally distributed data of phenoloxidase activity were analysed using ANOVA with post-hoc Tukey's HSD test. In dose-response experiments, effect of each particular dose of PHL was compared to PBS control using Dunnett's multiple comparisons test. Differences were considered statistically significant for p values < 0.05.

### Ethics statement

Anonymised human blood of blood groups A, B, O treated with natrium citrate was purchased from Transfusion and Tissue Department, The University Hospital Brno, Czech Republic. IRB approval for use of these samples is not requested. It was also confirmed by the university body.

## Supporting information

S1 Table Glycan array results. Screening of glycans printed on a microarray chip (Semiotik, Moscow; Slide number 10085636) via a standard manufacturer’s procedure. Results were calculated from hexaplicates.
(XLSX)

S2 Table Glycan array—Raw data. (XLSX)

S1 Fig Dose-dependence of PHL effect on ROS production. Whole blood was incubated with different doses of PHL and the subsequent production of ROS was measured luminometrically in the absence (without activator; white columns) or presence of the activator zymosan A (black columns). The presented data show the integral of ROS production in blood with PHL normalised to the integral of the reaction with PBS ± SD; * indicates significant difference (p < 0.05; Dunnett's test).
(TIF)

S2 Fig Dose-dependence of PHL effect on antimicrobial activity against *E*. *coli* K12 in human serum. Serum was incubated with different doses of PHL and mixed with bioluminescent bacteria *E*. *coli* K12. The bioluminescence was measured and evaluated as described in Experimental procedure. The results are expressed as difference between total measurement time (90 min) and time needed to reach threshold of bioluminescence ± SD; * indicates significant difference p < 0.05, ** p < 0.01 (Dunnett's test).
(TIF)
