# Supplementary figures and images for: Characterization of novel bangle lectin from Photorhabdus asymbiotica with dual sugar-binding specificity and its effect on host immunity
Source: PLoS Pathog. 2017 Aug 14;13(8):e1006564. doi: 10.1371/journal.ppat.1006564 (PMC5584973; doi:10.1371/journal.ppat.1006564)

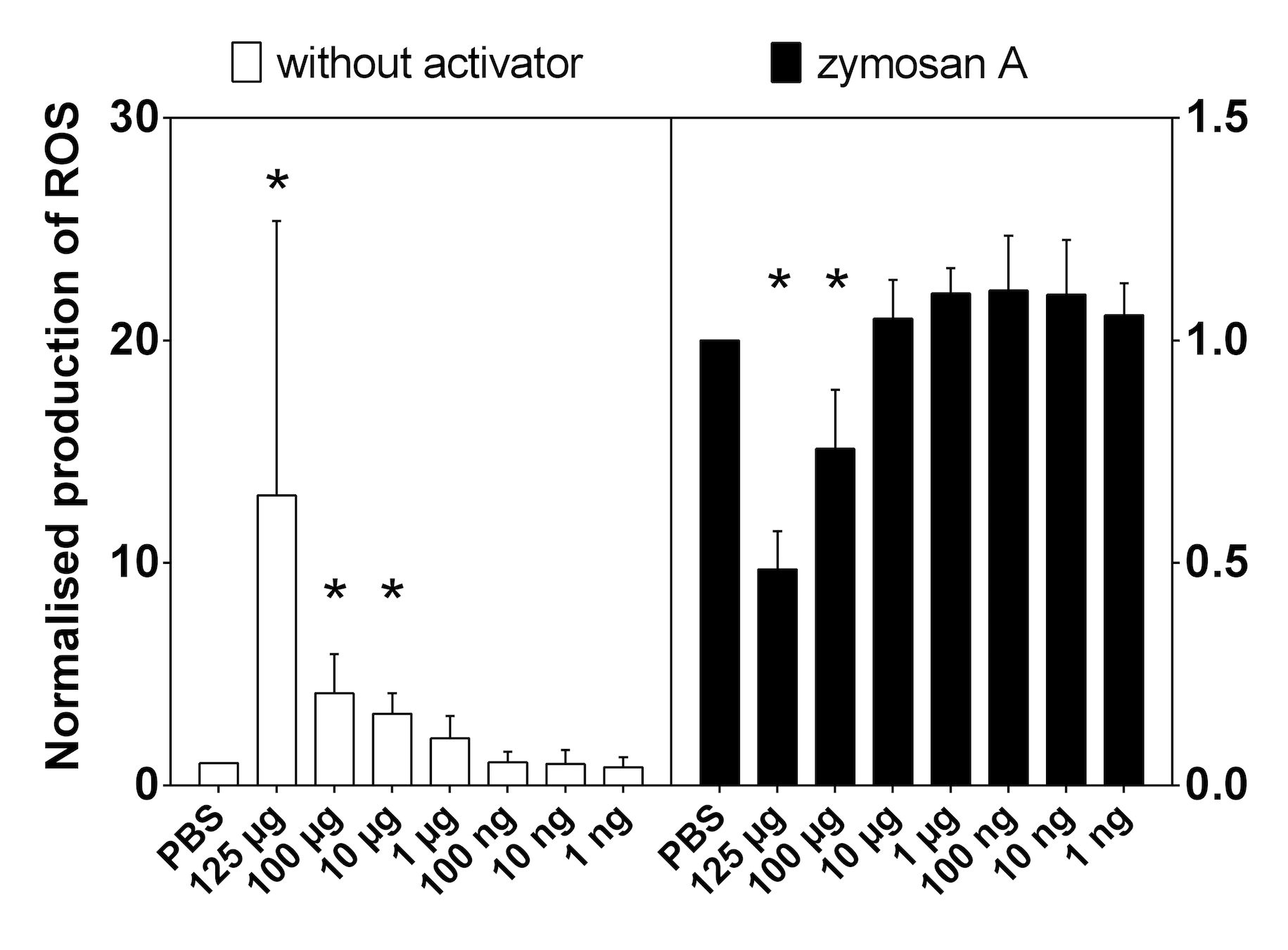

Supplement: S1 Fig — Whole blood was incubated with different doses of PHL and the subsequent production of ROS was measured luminometrically in the absence (without activator; white columns) or presence of the activator zymosan A (black columns). The presented data show the integral of ROS production in blood with PHL normalised to the integral of the reaction with PBS ± SD; * indicates significant difference (p < 0.05; Dunnett's test). (TIF) [file ppat.1006564.s003.tif]

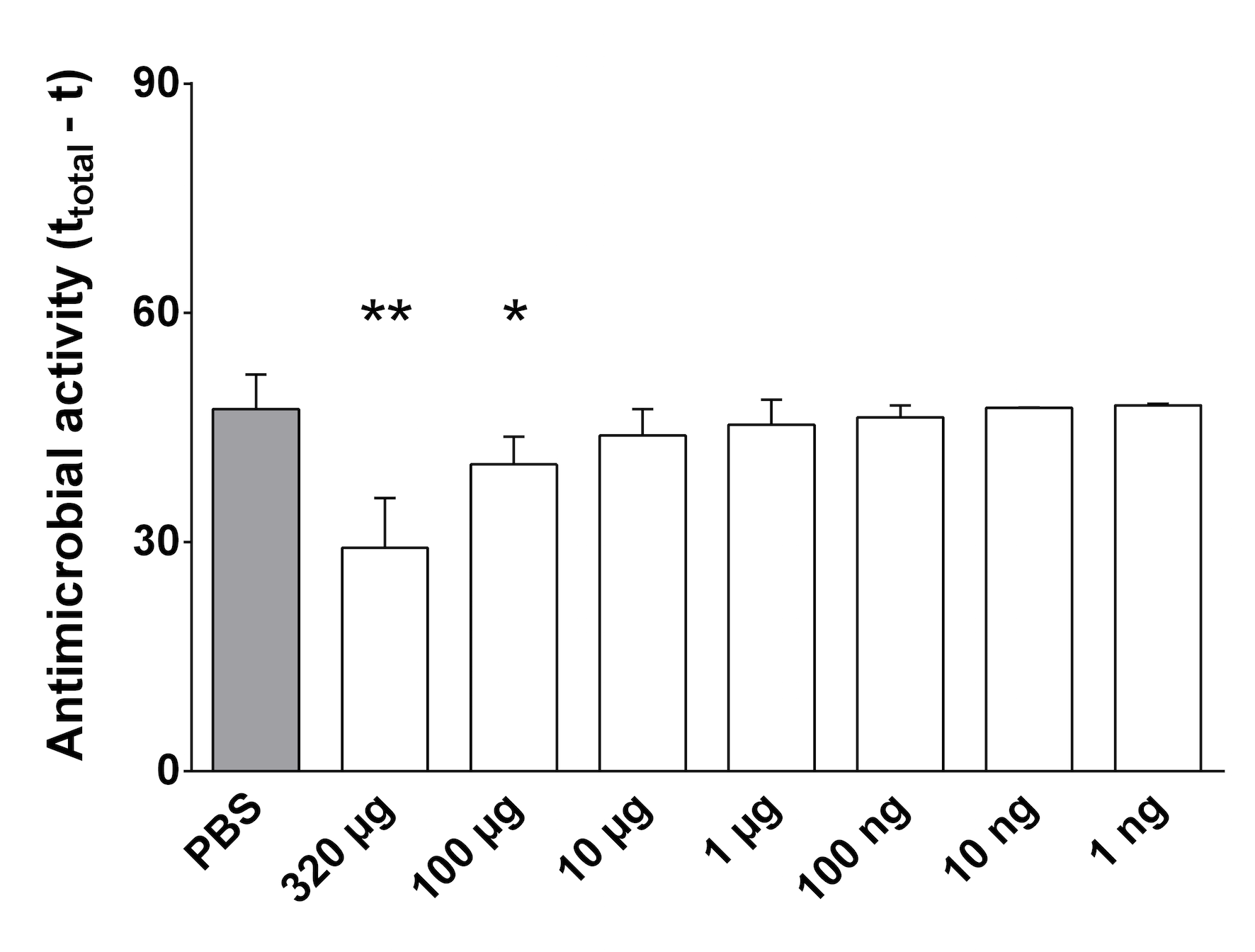

Supplement: S2 Fig — Serum was incubated with different doses of PHL and mixed with bioluminescent bacteria E. coli K12. The bioluminescence was measured and evaluated as described in Experimental procedure. The results are expressed as difference between total measurement time (90 min) and time needed to reach threshold of bioluminescence ± SD; * indicates significant difference p < 0.05, ** p < 0.01 (Dunnett's test). (TIF) [file ppat.1006564.s004.tif]
